# Who mixes with whom among men who have sex with men? Implications for modelling the HIV epidemic in southern India

**DOI:** 10.1016/j.jtbi.2014.04.005

**Published:** 2014-08-21

**Authors:** K.M. Mitchell, A.M. Foss, H.J. Prudden, Z. Mukandavire, M. Pickles, J.R. Williams, H.C. Johnson, B.M. Ramesh, R. Washington, S. Isac, S. Rajaram, A.E. Phillips, J. Bradley, M. Alary, S. Moses, C.M. Lowndes, C.H. Watts, M.-C. Boily, P. Vickerman

**Affiliations:** a London School of Hygiene and Tropical Medicine, London, UK; b Imperial College London, London, UK; c Karnataka Health Promotion Trust, Bangalore, India; d University of Manitoba, Winnipeg, MB, Canada; e St. John’s Research Institute, Bangalore, India; f CHARME-India Project, Bangalore, India; g Centre de recherche du CHU de Québec, Québec, QC, Canada; h Département de medicine sociale et preventive, Université laval, Québec, QC, Canada; i Institut national de santé publique du Québec, Québec, QC, Canada; j Public Health England, London, UK; k University of Bristol, Bristol, UK

**Keywords:** Mathematical model, Mixing matrix, Sexually transmitted infection, Disassortative mixing, Pre-exposure prophylaxis

## Abstract

In India, the identity of men who have sex with men (MSM) is closely related to the role taken in anal sex (insertive, receptive or both), but little is known about sexual mixing between identity groups. Both role segregation (taking only the insertive or receptive role) and the extent of assortative (within-group) mixing are known to affect HIV epidemic size in other settings and populations. This study explores how different possible mixing scenarios, consistent with behavioural data collected in Bangalore, south India, affect both the HIV epidemic, and the impact of a targeted intervention. Deterministic models describing HIV transmission between three MSM identity groups (mostly insertive *Panthis*/Bisexuals, mostly receptive *Kothis*/*Hijras* and versatile Double Deckers), were parameterised with behavioural data from Bangalore. We extended previous models of MSM role segregation to allow each of the identity groups to have both insertive and receptive acts, in differing ratios, in line with field data. The models were used to explore four different mixing scenarios ranging from assortative (maximising within-group mixing) to disassortative (minimising within-group mixing). A simple model was used to obtain insights into the relationship between the degree of within-group mixing, $R_{0}$ and equilibrium HIV prevalence under different mixing scenarios. A more complex, extended version of the model was used to compare the predicted HIV prevalence trends and impact of an HIV intervention when fitted to data from Bangalore. With the simple model, mixing scenarios with increased amounts of assortative (within-group) mixing tended to give rise to a higher $R_{0}$ and increased the likelihood that an epidemic would occur. When the complex model was fit to HIV prevalence data, large differences in the level of assortative mixing were seen between the fits identified using different mixing scenarios, but little difference was projected in future HIV prevalence trends. An oral pre-exposure prophylaxis (PrEP) intervention was modelled, targeted at the different identity groups. For intervention strategies targeting the receptive or receptive and versatile MSM together, the overall impact was very similar for different mixing patterns. However, for PrEP scenarios targeting insertive or versatile MSM alone, the overall impact varied considerably for different mixing scenarios; more impact was achieved with greater levels of disassortative mixing.

## Highlights

• Different mixing scenarios are explored for 3 groups of role-segregated MSM.
• Models show that the mixing scenario affects both *R*_0_ and endemic HIV prevalence.
• When models are fit to data, predicted HIV trends are unaffected by mixing.
• Impact of targeted (but not non-targeted) interventions can be affected by mixing.

## 1 Introduction

For men who have sex with men (MSM), taking the receptive role in anal sex carries a higher risk of acquiring HIV from an infected partner than taking the insertive role (Jin et al., 2010; Pinkerton et al., 2000; Vittinghoff et al., 1999). Mathematical models have shown that role segregation among MSM (adopting a fixed role – insertive or receptive – during all anal sex acts) can reduce the size of an HIV epidemic (Goodreau and Golden, 2007; Goodreau et al., 2005; Punyacharoensin et al., 2011; Van Druten et al., 1992; Wiley and Herschkorn, 1989). This is because when there is high role segregation, men consistently taking the receptive role have a high risk of acquiring infection but a low risk of transmitting, whereas when there is little role segregation, individuals acquiring HIV through receptive acts are more likely to transmit infection through taking the insertive role in some subsequent sexual encounters (Goodreau et al., 2005; Trichopoulos et al., 1988). Similarly, if individuals fluctuate between taking the insertive and receptive roles, this can increase HIV prevalence (Alam et al., 2010).

In some countries, MSM sexual identity is related to the role taken in anal sex (Goodreau et al., 2005), and this is seen in India, where *Kothis* and *Hijras* tend to take the receptive role, *Panthis* and Bisexuals usually take the insertive role, and Double Deckers are versatile, taking both insertive and receptive roles (Asthana and Oostvogels, 2001; Boyce, 2007; Phillips et al., 2008). Hence it is likely that considerable role segregation occurs among MSM in India. Reported behaviour does not map exactly onto identity groups however, with some insertive acts reported by *Kothis* and some receptive acts reported by Bisexuals and *Panthis* (Phillips et al., 2008). Limited data are currently available on mixing between these different identity groups in India. It is important to explore the implications of different possible mixing patterns between MSM groups in the southern Indian setting, since there is an extensive literature showing that mixing between different groups (stratified by age, race or levels of sexual activity) can have a large impact on HIV prevalence (Boily and Anderson, 1991; Garnett and Anderson, 1994; Hallett et al., 2007; Turner et al., 2004).

Disassortative mixing is defined as preferentially mixing with other groups rather than with your own group. For example, in heterosexual models, 100% disassortative mixing by gender is usually assumed, where men only have sex with women and vice versa. Disassortative mixing is also expected in role-segregated MSM populations, since those taking the receptive role must mix with those taking the insertive role. When there is disassortative mixing, there is usually a discrepancy in the number of sex acts reported by different groups (e.g. men usually report having more sex acts than women), so for modelling, the data have to be adjusted to allow the number of acts between different groups to ‘balance’. In models of heterosexual STI transmission, simple and transparent methods for balancing sex acts have been developed (Garnett and Anderson, 1994; Gupta et al., 1989; Hallett et al., 2007; Turner et al., 2004). However, balancing and determining mixing in models including MSM role segregation is more complex and a variety of methods have been used for different settings (Alam et al., 2010; Goodreau and Golden, 2007; Goodreau et al., 2005; Lou et al., 2009; Sullivan et al., 2012). These previous models have all divided the population into three groups: exclusively insertive, exclusively receptive, and versatile; none have allowed for occasional receptive acts by insertive MSM or any insertive acts by mainly receptive MSM.

In this paper, we develop several new mixing matrices (which determine the probabilities of mixing between different MSM groups), that all ensure balancing of insertive and receptive anal sex acts, for three MSM groups with different levels of insertive and receptive anal sex (note that in contrast to previous models, none of these groups have exclusively insertive or receptive sex). We parameterise these matrices with data from Bangalore in southern India, and explore how different mixing scenarios within this framework affect the level of overall mixing between groups, the $R_{0}$ for HIV, and the size of an HIV epidemic. We also explore the implications of these different mixing scenarios for estimating the impact of a targeted HIV intervention after fitting a dynamic HIV transmission model incorporating these mixing assumptions to data from Bangalore. The intervention explored is pre-exposure prophylaxis (PrEP), an oral antiretroviral medication which when taken daily by uninfected MSM has been shown to reduce their risk of acquiring HIV (Grant et al., 2011, 2010). Since PrEP is only delivered to, and directly affects, HIV negative individuals, it can be independently targeted at different population groups.

## 2 Methods

### 2.1 Data sources

The data were collected from MSM in Bangalore for evaluation of the *Avahan* intervention, an HIV prevention program targeted at vulnerable groups in six high prevalence states in India (Chandrasekaran et al., 2008). MSM were analysed in three groups, by the role taken in anal sex: *Kothis*/*Hijras* (KH; predominantly receptive), *Panthis*/Bisexuals (PB; predominantly insertive) and Double Deckers (DD; versatile). Data from Integrated Biological and Behavioural Assessment (IBBA) surveys in 2006 (Brahmam et al., 2008) and 2009 were used to calculate group-specific HIV prevalence, frequency of anal sex and condom use. Data from the 2006 IBBA survey on the identity of main MSM partners (for the subset of MSM who reported having a main partner) was used to inform the structure of the ‘setting plausible’ mixing matrix (Section 2.3). Historical condom use trends were based upon a statistical analysis of data from the 2006 IBBA survey on how long MSM had been having sex with men and how long they had always used condoms with male sex partners, which was used to reconstruct the trend in consistent condom use over time (Lowndes et al., 2010). Data from a special behavioural survey (SBS) carried out in 2006 (Phillips et al., 2008) were used to calculate the percentage of anal sex acts that are insertive for each group. The size of the KH population in Bangalore was estimated from mapping surveys carried out by non-governmental organisations (Bill & Melinda Gates Foundation, 2008), and the size of the DD population estimated from their frequency relative to the numbers of KH in the IBBA surveys. Total MSM population size was estimated from confidential Polling Booth Surveys (PBS) conducted among the general population in neighbouring districts (Bellary, Belgaum and Mysore (Lowndes et al., 2012)). The size of the PB population could thus be estimated by subtracting the sizes of the KH and DD populations from the total population size. Note that there is considerable uncertainty in these metrics, particularly the population sizes, which is taken into account in the analyses.

### 2.2 Balancing overall number of sex acts

Mixing matrices were developed describing the mixing between three MSM groups: KH, PB and DD. Mixing was determined within the constraints of group-specific data on group size, frequency of anal sex, and proportion of acts which are insertive.

The overall numbers of insertive ($Ins_{i}$) and receptive ($Rec_{i}$) acts for each identity group were calculated as:

(1) $$Ins_{i}=N_{i}c_{i}x_{i}$$

(2) $$Rec_{i}=N_{i}c_{i}(1-x_{i})$$

where $N_{i}$=population size, $c_{i}$=number of anal sex acts per month per man, and $x_{i}$=proportion of acts which are insertive, for each identity group (i=KH, PB, DD).

Balancing was achieved by altering the PB population size (for which no data are available) to give an equal number of insertive and receptive acts across the whole population.

If the total numbers of insertive and receptive acts in the whole population balance, then:

(3) $$Ins_{KH}+Ins_{PB}+Ins_{DD}=Rec_{KH}+Rec_{PB}+Rec_{DD}$$

Substituting (1) and (2) into (3) and re-arranging gives

(4) $$N_{PB}=\frac{N_{KH}c_{KH}(1-2x_{KH})+N_{DD}c_{DD}(1-2x_{DD})}{c_{PB}(2x_{PB}-1)}$$

### 2.3 Mixing matrices

Having balanced the total number of insertive and receptive acts, we then distributed the insertive and receptive acts between the different groups within data constraints, using one of four different mixing scenarios: (i) maximum assortative (maximising within-group mixing while maintaining the calculated numbers of insertive and receptive acts for each group, which prevents fully assortative mixing); (ii) setting plausible (in which DD have maximum within-group mixing while the other groups mix disassortatively; this scenario was developed following discussions with local MSM and analysis of data on the reported identity of main MSM partners); (iii) proportionate (in which receptive acts are distributed in proportion to the number of insertive acts offered by each group; note that this does not give rise to overall proportionate (random) mixing in the population due to the different groups having differing levels of insertive and receptive sex), and (iv) disassortative (minimising within-group mixing while maintaining the calculated numbers of insertive and receptive acts for each group, which prevent fully disassortative mixing). Note that the name of each matrix refers to the rules used to distribute the sex acts within the data constraints—it does not reflect the resulting overall pattern of mixing, which is also influenced by the relative amounts of insertive and receptive sex that each group has. The derivation of each mixing matrix is presented in Supplementary materials, together with details on how the number of sex acts and overall mixing probabilities were calculated.

### 2.4 Calculating the degree of overall mixing (Q)

For each mixing scenario, and for each parameter combination considered, the overall degree of mixing was calculated from the overall mixing matrix (Supplementary materials) using *Q* (Gupta et al., 1989), which assesses within-group mixing. For a matrix with n groups, given the probabilities ($\rho_{i,j}$) that group i mixes with group *j*, *Q* is calculated as:

$$Q=\frac{\sum_{i=j}\rho_{\;i,j}-1}{n-1}$$

A *Q* of 1 indicates completely assortative mixing, 0 indicates proportionate mixing, and a negative *Q* value indicates disassortative mixing. When three groups are used (n=3), a *Q* of −0.5 indicates completely disassortative mixing.

### 2.5 Analyses using simple, unfitted model

A simple model (which does not include acute or late-stage HIV, nor any condom use trends) was developed and used to calculate the basic reproductive number ($R_{0}$) and equilibrium prevalence of HIV for the different mixing scenarios, and these were compared with each other and with *Q* (the degree of mixing) for multiple parameter combinations. The proportion of new infections from each group when HIV reached endemic equilibrium prevalence was also calculated to estimate the contribution of each group to HIV transmission. For these initial analyses, the model was not fit to HIV prevalence data. This allowed direct comparisons to be made between the four mixing scenarios for each parameter combination.

#### 2.5.1 Description of the simple model

A deterministic compartmental SI model was developed to describe the transmission of HIV between and amongst the three MSM groups (KH, DD and PB). The MSM are further divided into susceptible and infectious compartments, denoted by $S_{i}$ and $I_{i}$ (where i=KH, DD or PB), and the total size of each identity group, $N_{i}$, is given by $S_{i}+I_{i}$. Susceptible MSM in each identity group ($S_{i}$), become infected at a per-person time dependent rate $\lambda_{i}$, and move into the infected compartment ($I_{i}$). The force of infection, $\;\lambda_{i}$, is a function of the number of anal sex acts per month per man ($c_{i}$), the proportion of acts which are insertive ($x_{i}$), the probability that HIV is acquired from an infectious partner per receptive act ($\beta_{rec}$), the relative risk of acquiring HIV from an insertive compared with a receptive sex act (κ), the mixing matrices ($\rho_{Ins\;}$ and $\rho_{Rec}$), and the prevalence of HIV in each of the groups they are mixing with (given by $I_{j}/N_{j}$). MSM in both susceptible and infected compartments cease sexual activity with other men for non-HIV related reasons and leave the MSM population at a constant per-person rate $\mu_{i}$. Additionally, MSM in the infected compartments leave the population as they develop AIDS, at which point they are assumed to cease sexual activity, at a constant per-person rate *α*. New sexually active MSM join the population at a constant rate $\mu_{i}N_{i}^{0}$ where $N_{i}^{0}$ is assumed to be the initial size of the MSM in group i, prior to the introduction of HIV into the population.

The rate of change of the number of susceptible ($S_{i}$) and HIV-infected ($I_{i}$) MSM in each group (i=KH, DD, PB) is given by the following system of differential equations

$$\{\begin{array}{l} \frac{dS_{i}}{dt}=\mu_{i}N_{i}^{0}-\lambda_{i}S_{i}-\mu_{i}S_{i}\;\\ \frac{dI_{i}}{dt}=\lambda_{i}S_{i}-\mu_{i}I_{i}-\;\alpha I_{i} \end{array}$$

The force of infection is given by

$$\lambda_{i}(t)=\sum_{j=1}^{n}\left(\left(c_{i}x_{i}\rho_{Ins,i,j}\kappa \beta_{rec}\frac{I_{j}}{N_{j}}\right)+\left(c_{i}(1-x_{i})\rho_{Rec,i,j}\beta_{rec}\frac{I_{j}}{N_{j}}\right)\right)$$

where *n* is the number of groups in the model (*n*=3 for all analyses here).

In order to maintain balancing of sex acts over time, $c_{PB}$ is recalculated at each time step using the following equation, which is re-arranged from Eq. (4)

$$c_{PB}=\frac{N_{KH}c_{KH}(1-2x_{KH})+N_{DD}c_{DD}(1-2x_{DD})}{N_{PB}(2x_{PB}-1)}$$

The mixing matrix is also re-calculated at each time step, using the methods described in Supplementary materials, using current population sizes and contact rates for the three groups.

We repeated analyses with the simple model making an alternative assumption that the sizes of the total MSM population and each MSM group (KH, DD and PB) remain constant over time, by replacing all of those ceasing sexual activity and leaving the population (due to AIDS or other reasons) with sexually active MSM of the same identity group newly entering the susceptible population.

#### 2.5.2 Calculating the basic reproductive number ($R_{0}$) for the simple model

The basic reproductive number ($R_{0}$) is the average number of secondary HIV cases arising from the introduction of a single infectious individual into a completely susceptible population, and is mathematically defined as the spectral radius of the next generation operator (Diekmann et al., 1990; van den Driessche and Watmough, 2002). Using an approach in van den Driessche and Watmough (2002) to calculate $R_{0}$ for the simple model, at the disease-free equilibrium ($\xi^{0}=(S_{KH}^{0},I_{KH}^{0},\;S_{DD}^{0},I_{DD}^{0},S_{PB}^{0},I_{PB}^{0},)=(N_{KH}^{0},0,N_{DD}^{0},0,N_{PB}^{0},0))$, the *F* matrix for the rate of new infections coming into each compartment, and the V matrix for all other transitions into and out of compartments are given by

$$F=\left(\begin{array}{ccc} c_{KH}xi_{KH}\kappa \beta_{R}\rho_{Ins,KH,KH}+c_{KH}(1-x_{KH})\beta_{R}\rho_{Rec,KH,KH} & \frac{N_{KH}^{0}c_{KH}x_{KH}\kappa \beta_{R}\rho_{Ins,KH,DD}+c_{KH}(1-x_{KH})\beta_{R}\rho_{Rec,KH,DD}}{N_{DD}^{0}} & \frac{N_{KH}^{0}c_{KH}x_{KH}\kappa \beta_{R}\rho_{Ins,KH,PB}+c_{KH}\left(1-x_{KH}\right)\beta_{R}\rho_{Rec,KH,PB}}{N_{PB}^{0}}\\ \frac{N_{DD}^{0}c_{DD}x_{DD}\kappa \beta_{R}\rho_{Ins,DD,KH}+c_{DD}(1-x_{DD})\beta_{R}\rho_{Rec,DD,KH}}{N_{KH}^{0}} & c_{DD}x_{DD}\kappa \beta_{R}\rho_{Ins,DD,DD}+c_{DD}(1-x_{DD})\beta_{R}\rho_{Rec,DD,DD} & \frac{N_{DD}^{0}c_{DD}x_{DD}\kappa \beta_{R}\rho_{Inc,DD,PB}+c_{DD}(1-x_{DD})\beta_{R}\rho_{Rec,DD,PB}}{N_{PB}^{0}}\\ \frac{N_{PB}^{0}c_{PB}x_{PB}\kappa \beta_{R}\rho_{Inc,PB,KH}+c_{PB}(1-x_{PB})\beta_{R}\rho_{Rec,PB,KH}}{N_{KH}^{0}} & \frac{N_{PB}^{0}c_{PB}x_{PB}\kappa \beta_{R}\rho_{Inc,PB,DD}+c_{PB}(1-x_{PB})\beta_{R}\rho_{Rec,PB,DD}}{N_{DD}^{0}} & c_{PB}x\kappa \beta_{R}\rho_{Inc,PB,PB}+c_{PB}(1-x_{PB})\beta_{R}\rho_{Rec,PB,PB} \end{array}\right)V=\left(\begin{array}{ccc} \alpha +\mu_{KH} & 0 & 0\\ 0 & \alpha +\mu_{DD} & 0\\ 0 & 0 & \alpha +\mu_{PB} \end{array}\right).$$

The next generation matrix is given by

$$FV^{-1}=\begin{array}{ccc} \frac{c_{KH}x_{KH}\kappa \beta_{R}\rho_{Ins,KH,KH}+c_{KH}(1-x_{KH})\beta_{R}\rho_{Rec,KH,KH}}{\alpha +\mu_{KH}} & \frac{N_{KH}^{0}c_{KH}x_{KH}\kappa \beta_{R}\rho_{Ins,KH,DD}+c_{KH}(1-x_{KH})\beta_{R}\rho_{Rec,KH,DD}}{N_{DD}^{0}(\alpha +\mu_{DD})} & \frac{N_{KH}^{0}c_{KH}x\kappa \beta_{R}\rho_{Ins,KH,PB}+c_{KH}(1-x_{KH})\beta_{R}\rho_{Rec,KH,PB}}{N_{PB}^{0}(\alpha +\mu_{PB})}\\ \frac{N_{DD}^{0}c_{DD}x_{DD}\kappa \beta_{R}\rho_{Ins,DD,KH}+c_{DD}(1-x_{DD})\beta_{R}\rho_{Rec,DD,KH}}{N_{KH}^{0}(\alpha +\mu_{KH})} & \frac{c_{DD}x_{DD}\kappa \beta_{R}\rho_{Ins,DD,DD}+c_{DD}(1-x_{DD})\beta_{R}\rho_{Rec,DD,DD}}{\alpha +\mu_{DD}} & \frac{N_{DD}^{0}c_{DD}x_{DD}\kappa \beta_{R}\rho_{Ins,DD,PB}+c_{DD}(1-x_{DD})\beta_{R}\rho_{Rec,DD,PB}}{N_{PB}^{0}(\alpha +\mu_{PB})}\\ \frac{N_{PB}^{0}c_{PB}x\kappa \beta_{R}\rho_{Ins,PB,KH}+c_{PB}(1-x_{PB})\beta_{R}\rho_{Rec,PB,KH}}{N_{KH}^{0}(\alpha +\mu_{KH})} & \frac{N_{PB}^{0}c_{PB}x\kappa \beta_{R}\rho_{Ins,PB,DD}+c_{PB}(1-x_{PB})\beta_{R}\rho_{Rec,PB,DD}}{N_{DD}^{0}(\alpha +\mu_{DD})} & \frac{c_{PB}x\kappa \beta_{R}\rho_{Ins,PB,PB}+c_{PB}(1-x_{PB})\beta_{R}\rho_{Rec,PB,PB}}{\alpha +\mu_{PB}} \end{array}=\left(\begin{array}{ccc} R_{KH,KH}\; & R_{KH,DD} & R_{KH,PB}\\ R_{DD,KH} & R_{DD,DD} & R_{DD,PB}\\ R_{PB,KH} & R_{PB,DD} & R_{PB,PB} \end{array}\right).$$

Here, $R_{i,j}$ for (i,j)=KH, PB and DD are the expected number of group i individuals infected by group j individuals when a single newly infected individual in group j enters the disease-free population at equilibrium.

It follows that the basic reproductive number for the simple model is given by $R_{0}=\rho (FV^{-1})$, which is the dominant eigenvalue of the next generation matrix (*ρ* denotes the spectral radius). Here, the structure of the next generation matrix hinders the derivation of an analytic expression for $R_{0}$. Therefore we calculate $R_{0}$ numerically using code in C++ which makes use of the Unsymmeig routine from Press et al. (2007), computing the eigenvalues of a real nonsymmetric matrix by reduction to Hessenberg form, followed by *QR* iteration. Local stability of the disease-free equilibrium for the simple model when $R_{0}$<1 follows from Theorem 2 in van den Driessche and Watmough (2002).

#### 2.5.3 Calculating the equilibrium prevalence of HIV for the simple model

The endemic equilibrium for simple model in terms of the group force of infection ($\lambda_{i}^{⁎}$) is given by

$$\xi^{⁎}=\{\begin{array}{c} S_{KH}^{⁎}=\frac{\mu_{KH}N_{KH}^{0}}{\lambda_{KH}^{⁎}+\mu_{KH}},\;I_{KH}^{⁎}=\frac{\mu_{KH}N_{KH}^{0}\lambda_{KH}^{⁎}}{\left(\alpha +\mu_{KH})(\lambda_{KH}^{⁎}+\mu_{KH}\right)}\\ S_{DD}^{⁎}=\frac{\mu_{DD}N_{DD}^{0}}{\lambda_{DD}^{⁎}+\mu_{DD}},\;I_{DD}^{⁎}=\frac{\mu_{DD}N_{DD}^{0}\lambda_{DD}^{⁎}}{\left(\alpha +\mu_{DD})(\lambda_{DD}^{⁎}+\mu_{DD}\right)}\\ S_{PB}^{⁎}=\frac{\mu_{PB}N_{PB}^{0}}{\lambda_{PB}^{⁎}+\mu_{PB}},\;I_{PB}^{⁎}=\frac{\mu_{PB}N_{PB}^{0}\lambda_{PB}^{⁎}}{\left(\alpha +\mu_{PB})(\lambda_{PB}^{⁎}+\mu_{PB}\right)} \end{array}$$

where $S_{i}^{⁎}$ is the number of susceptible MSM, and $I_{i}^{⁎}$ the number of infected MSM, in group i at endemic equilibrium.

Due to the complexity of the model, it was not possible to explicitly derive algebraically tractable solutions for the endemic equilibrium. We used numerical simulations to demonstrate the existence and uniqueness of the endemic equilibrium. The equilibrium HIV prevalence was calculated for each mixing matrix using numerical simulation of the deterministic transmission model, starting with 1% of each group in the infected compartment ($I_{i}$) and the remainder in the susceptible compartment ($S_{i}$). For each parameter set, and each mixing scenario in turn, the equations were solved numerically using an Euler algorithm and a time step of 0.001 years, which continued until HIV prevalence reached endemic equilibrium (defined as changing by less than 0.01% per year). The solution profile for the infected population using various initial conditions for $R_{0}$<1 and $R_{0}$>1 for all mixing scenarios shows convergence to the disease-free and a unique endemic equilibrium respectively (Supplementary Fig. S1), suggesting that a unique endemic equilibrium exists for this model.

#### 2.5.4 Contribution of each group to incidence at equilibrium

To see the contribution of each MSM group to the spread of HIV, we calculated the proportion of new infections which are generated by each group (i.e. proportion of newly infected who are infected by members of that group) when HIV was at endemic equilibrium. At the endemic equilibrium in the numerical simulations, the proportion of new HIV infections coming from each group was calculated as follows:

$$\frac{\sum_{i=1}^{3}\left(\left(c_{i}x_{i}\kappa \beta_{rec}\rho_{Inc,i,j}+c_{i}(1-x_{i})\beta_{rec}\rho_{Rec,i,j}\right)S_{i}^{⁎}\right)}{\sum_{j=1}^{3}\left(\sum_{i=1}^{3}\left(\left(c_{i}x_{i}\kappa \beta_{rec}\rho_{Ins,i,j}+c_{i}(1-x_{i})\beta_{rec}\rho_{Rec,i,j}\right)S_{i}^{⁎}\right)\right)}$$

where $S_{i}^{⁎}$ is the proportion of susceptible MSM in group i at endemic equilibrium.

#### 2.5.5 Model parameters and initial analyses using simple model

Uncertainty ranges for behavioural parameters were obtained from data for Bangalore or neighbouring areas if data for Bangalore were not available (Section 2.1), and biological parameters were obtained from the literature (all given in Supplementary Table S1). For analyses involving the simple model, all behavioural and biological parameters were varied within the ranges shown in Supplementary Table S1. Parameters were sampled from their ranges 1000 times using Latin Hypercube sampling (Blower and Dowlatabadi, 1994), assuming a uniform distribution for each parameter. To check whether the assumed distribution made a difference, parameters were then resampled 1000 times, first assuming a triangular distribution for each parameter (using the mode and range given in Supplementary Table S1), and then assuming a normal distribution for each parameter (using the mean and standard deviation given in Supplementary Table S1). Parameter sets which gave rise to a data-plausible overall MSM population size (between 8501 and 122,600) were used to calculate *Q*, $R_{0}$ and the equilibrium HIV prevalence using each of the different mixing scenarios, using the calculations in Sections 2.4, 2.5.2 and 2.5.3. $R_{0}$ was estimated using data at the start of the epidemic, and *Q* was calculated at equilibrium prevalence. All of these analyses were repeated using the alternative model assumption of population sizes remaining constant over time.

### 2.6 Analyses using more complex model, fitted to data

A more complex model was developed which included three stages of HIV infection (to account for the increased transmission probabilities associated with early and late-stage HIV infection) and time-varying condom use. This model was fitted to HIV prevalence data, and model fits were used to compare the differences in mixing patterns and HIV prevalence trends for different mixing scenarios, the impact of a targeted PrEP intervention from 2015, and the effective reproductive number (R) with and without PrEP.

#### 2.6.1 Complex model structure

The complex model is an extension of the simple HIV model which includes three stages of HIV infection, acute, chronic and pre-AIDS, where HIV-positive MSM in the acute and pre-AIDS stages are more highly infectious than those in the chronic stage. As in the simple model, susceptible MSM ($S_{i}$), become infected at a rate $\;\lambda_{i}$, which is determined by the number and type (insertive or receptive) of sex acts they have, the per-act probabilities of acquiring HIV from an HIV-infected partner associated with receptive and insertive sex acts, mixing patterns and HIV prevalence of partners in different groups. Additionally in this model, infection rates are affected by time-varying condom use (which is assumed to be the same across all groups), and by the stage of HIV infection of HIV-positive partners. Once infected, MSM enter the acute HIV infection compartment ($I_{i,1}$), where they remain for 1/$\gamma_{1}$ years before moving into the chronic infection stage ($I_{i,2}$). MSM stay in the chronic infection stage for on average 1/$\gamma_{2}\;$ years before moving into the pre-AIDS stage ($I_{i,3}$), which they leave at a rate α as they develop AIDS and cease sexual activity. As in the simple model, MSM leave all compartments at a constant per-person rate $\mu_{i}$ as they cease sexual activity with other men for non-HIV related reasons, and are recruited into the population at a constant rate $\mu_{i}N_{i}^{0}$. As in the simple model, the contact rate for the PB group and the mixing parameters were re-calculated at each timestep to ensure balancing of sex acts between the different groups. The rates of change of the number of MSM who are susceptible ($S_{i}$), have acute ($I_{i,1}$), chronic ($I_{i,2}$) or pre-AIDS ($I_{i,3}$) HIV infection in each MSM group (i=KH, DD, PB) are given by the following system of equations

$$\{\begin{array}{l} \frac{dS_{i}}{dt}=\mu_{i}N_{i}^{0}-S_{i}(\lambda_{i}+\mu_{i})\;\\ \frac{dI_{i,1}}{dt}=\lambda_{i}S_{i}-I_{i,1}(\mu_{i}+\gamma_{1})\;\\ \frac{dI_{i,2}}{dt}=\gamma_{1}I_{i,1}-I_{i,2}(\mu_{i}+\gamma_{2})\\ \frac{dI_{i,3}}{dt}=\gamma_{2}I_{i,2}-I_{i,3}(\mu_{i}+\alpha ).\; \end{array}$$

The force of infection is given by,

$$\lambda_{i}(t)=\sum_{j=1}^{n}(1-e_{C}f_{C}(t))(\left(c_{i}x_{i}\rho_{Ins,i,j}\kappa \left(\beta_{rec,1}\;\frac{I_{j,1}}{N_{j}}+\beta_{rec,2}\frac{I_{j,2}}{N_{j}}+\beta_{rec,3}\;\frac{I_{j,3}}{N_{j}}\right)\;\right)+\left(c_{i}(1-x_{i})\rho_{Rec,i,j}\left(\beta_{rec,1}\;\frac{I_{j,1}}{N_{j}}+\beta_{rec,2}\;\frac{I_{j,2}}{N_{j}}+\beta_{rec,3}\;\frac{I_{j,3}}{N_{j}}\right)\right))$$

where $e_{C}$ is condom efficacy against HIV, $\;f_{C}(t)$ is the proportion of sex acts in which condoms are used correctly and $\beta_{rec,j}$ is the per-act transmission from an individual in infection stage $I_{i,j}$. Based on statistical analyses reconstructing historical condom use trends (Lowndes et al., 2010), condom use was assumed to change over time in a linear piecewise manner according to the following equation

$$f_{C}(t)=\{\begin{array}{ll} \pi_{1}, & t\geq 2006\\ (t-t_{0})\Delta_{c},\; & t_{0}<t<2006,\;\pi >\pi_{0}\\ \pi_{0},\; & \mathrm{otherwise} \end{array}$$

where $\pi_{1}$ is the proportion of sex acts in which condoms are used correctly in the year 2006 (from 2006 IBBA survey data (Brahmam et al., 2008)), $t_{0}$ is the year in which condom use would have been zero when reconstructed condom use trends are extrapolated backwards in time, $\Delta_{c}$ is the yearly increase in the proportion of sex acts in which condoms are used correctly (Lowndes et al., 2010) and $\pi_{0}$ is the initial baseline level of condom use assumed in the population prior to the recently recorded increase.

We repeated analyses with the complex model making the alternative assumption that the sizes of the total MSM population and each MSM group remain constant over time, with all those leaving the population, due to developing AIDS or other causes, being replaced with new susceptible MSM of the same identity group.

#### 2.6.2 Model fitting and analyses of model fits

The complex model was fitted to HIV prevalence data for the three MSM groups from 2006 and 2009, using each mixing scenario in turn. All of the behavioural and biological parameters (detailed in Supplementary Table S1) were sampled from their ranges 1 million times using Latin Hypercube sampling, assuming a uniform distribution for each parameter. All parameter sets producing HIV prevalences within the range for each group in both 2006 and 2009, and population sizes for the KH and DD groups and the total MSM population within the ranges estimated in 2011 were retained as model fits. For these fits *Q*, within-group mixing, and overall and group-specific HIV prevalence over the period 1985–2030 were recorded.

#### 2.6.3 Comparing the impact of a targeted PrEP intervention

In order to test whether the choice of mixing scenario affects the predicted impact of a targeted intervention, we looked at a hypothetical PrEP intervention. PrEP was modelled against a background of reduced condom use to ensure that any differences in the effect of a PrEP intervention would be clearly seen. Condom use was assumed to drop linearly between 2010 and 2015, down to a level 20% (absolute difference) below the 2010 level, remaining constant thereafter. This fall in condom use might occur due to intervention fatigue or reduced perception of risk as HIV prevalence falls, but has not been observed in this setting to date.

PrEP was introduced in the model in 2015 without any gradual rollout phase. Incorporating PrEP in the model results in the following force of infection

$$\lambda_{i}(t)=\sum_{j=1}^{n}\left({\tilde{p}}_{i}(1-e_{C}f_{C}(t))(1-e_{P}\right)+(1-{\tilde{p}}_{i})(1-e_{C}f_{C}(t)))\times (\left(c_{i}x_{i}\rho_{Ins,i,j}\kappa \left(\beta_{rec,1}\;\frac{I_{j,1}}{N_{j}}+\beta_{rec,2}\;\frac{I_{j,2}}{N_{j}}+\beta_{rec,3}\;\frac{I_{j,3}}{N_{j}}\right)\right)+\left(c_{i}(1-x_{i})\rho_{Rec,i,j}\left(\beta_{rec,1}\;\frac{I_{j,1}}{N_{j}}+\beta_{rec,2}\;\frac{I_{j,2}}{N_{j}}+\beta_{rec,3}\;\frac{I_{j,3}}{N_{j}}\right)\;\right))$$

where ${\tilde{p}}_{i}$ is the proportion of MSM in group i who are using PrEP and $e_{P}$ is the effectiveness of PrEP in preventing HIV infection.

PrEP effectiveness – which combines PrEP efficacy and adherence – was assumed to be 42%, as measured in the iPrEx trial (Grant et al., 2011, 2010). For an optimistic high-adherence strategy, PrEP effectiveness was assumed to be 92%, based on trial findings for individuals with detectable treatment drug in their blood (Grant et al., 2011). Coverage of the target group(s) (i.e. the percentage of sex acts of susceptible MSM covered by the PrEP intervention) was assumed to be 30% or 60% when using a realistic effectiveness of 42%. For the optimistic high-adherence strategy, 90% coverage was assumed. The optimistic strategy was included to see whether differences emerged between the predictions for the different mixing scenarios under high intervention impact, assuming that any such differences would be greater and more easily observed with greater impact. PrEP was targeted separately at each of the three MSM identity groups, at the KH and DD groups together (because these groups are more likely to be in contact with current MSM-specific interventions than PB), and then at all three groups simultaneously.

Intervention impact was measured by summing up the total number of new infections occurring between 2015 and 2030 with and without the PrEP intervention included, and taking the difference to calculate the number of infections averted by the PrEP intervention. This was expressed as a percentage of the number of infections that would have occurred in the absence of PrEP (percentage of infections averted).

#### 2.6.4 Calculating the effective reproductive number (R) for the complex model

We define the effective reproductive number, R, as the number of secondary cases arising from a single index case in a completely susceptible population in the presence of an intervention (here, the intervention is condom use and/or PrEP).

Proceeding in the same way as we calculated $R_{0}$ for the simple model, R was calculated at the disease-free equilibrium using the approach in van den Driessche and Watmough (2002). The simplified next generation matrix is given as follows:

$$FV^{-1}=\left(\begin{array}{ccc} \frac{a_{1}b_{1}d_{1}}{\gamma_{1}+\mu_{KH}} & \frac{N_{KH\;KH}^{0}a_{1}b_{2}d_{2}}{N_{DD}^{0}(\gamma_{1}+\mu_{DD})} & \frac{N_{KH}^{0}a_{1}b_{3}d_{3}}{N_{PB}^{0}(\gamma_{1}+\mu_{PB)}}\\ \frac{N_{DD}^{0}a_{2}b_{1}d_{4}}{N_{KH}^{0}(\gamma_{1}+\mu_{KH})} & \frac{a_{2}b_{2}d_{5}}{\gamma_{1}+\mu_{DD}} & \frac{N_{DD}^{0}a_{2}b_{3}d_{6}}{N_{PB}^{0}(\gamma_{1}+\mu_{PB})}\\ \frac{N_{PB}^{0}a_{3}b_{1}d_{7}}{N_{KH}^{0}(\gamma_{1}+\mu_{KH})} & \frac{N_{PB}^{0}a_{3}b_{2}d_{8}}{N_{DD}^{0}(\gamma_{1}+\mu_{DD})} & \frac{a_{3}b_{3}d_{9}}{\gamma_{1}+\mu_{PB}} \end{array}\right)$$

Here, $a_{1}=c_{KH}(e_{c}f_{c}-1)(e_{p}{\tilde{p}}_{KH})\;$, $a_{2}=c_{DD}(e_{c}f_{c}-1)(e_{p}{\tilde{p}}_{DD})$, $a_{3}=c_{PB}(e_{c}f_{c}-1)(e_{p}{\tilde{p}}_{PB})$, $b_{1}=\beta_{rec,1}+(\gamma_{1}(\beta_{rec,3}\gamma_{2}+\beta_{rec,2}(\alpha +\mu_{KH}))/(\alpha +\mu_{KH})(\gamma_{2}+\mu_{KH}))$, $b_{2}=\beta_{rec,1}+(\gamma_{1}(\beta_{rec,3}\gamma_{2}+\beta_{rec,2}(\alpha +\mu_{DD}))/(\alpha +\mu_{DD})(\gamma_{2}+\mu_{DD}))$, $b_{3}=\beta_{rec,1}+(\gamma_{1}(\beta_{rec,3}\gamma_{2}+\beta_{rec,2}(\alpha +\mu_{PB}))/(\alpha +\mu_{PB})(\gamma_{2}+\mu_{PB}))$, $d_{1}=x_{KK}(\kappa \rho_{Ins,KH,KH}-\rho_{Rec,KH,KH})+\rho_{Rec,KH,KH}$, $\;d_{2}=x_{KK}(\kappa \rho_{Ins,KH,DD}-\rho_{Rec,KH,DD})+\rho_{Rec,KH,DD}$, $d_{3}=x_{KK}(\kappa \rho_{Ins,KH,PB}-\rho_{Rec,KH,PB})+\rho_{Rec,KH,PB}$, $\;d_{4}=x_{DD}(\kappa \rho_{Ins,DD,KH}-\rho_{Rec,DD,KH})+\rho_{Rec,DD,KH}$,$\;d_{5}=x_{DD}(\kappa \rho_{Ins,DD,DD}-\rho_{Rec,DD,DD})+\rho_{Rec,DD,DD}$, $d_{6}=x_{DD}(\kappa \rho_{Ins,DD,PB}-\rho_{Rec,DD,PB})+\rho_{Rec,DD,PB}$, $d_{7}=x_{PB}(\kappa \rho_{Ins,PB,KH}-\rho_{Rec,PB,KH})+\rho_{Rec,PB,KH}$, $d_{8}=x_{PB}(\kappa \rho_{Ins,PB,DD}-\rho_{Rec,PB,DD})+\rho_{Rec,PB,DD}$ and$\;d_{9}=x_{PB}(\kappa \rho_{Ins,PB,PB}-\rho_{Rec,PB,PB})+\rho_{Rec,PB,PB}$.

As before, the effective reproductive number is given by $R=\rho (FV^{-1})$ and is calculated using a numerical algorithm coded in C++, for scenarios with condom use and with or without PrEP included. Local stability of the disease-free equilibrium for the complex model system when R<1 also follows from van den Driessche and Watmough (2002).

## 3 Results

### 3.1 Analyses using simple, unfitted model

Out of the 1000 parameter sets selected from uniform parameter ranges, 595 gave a plausible total MSM population size when receptive and insertive sex acts were balanced. These 595 parameter sets were used in Sections 3.1.1–3.1.4, where all comparisons are between matched runs which differ only in the mixing scenario used. Analyses using parameters drawn from triangular or normal distributions gave qualitatively similar results—differences are noted where relevant. Analyses using the assumption of a constant population size also gave qualitatively similar results.

#### 3.1.1 Effect of mixing scenario upon overall mixing (*Q*)

*Q* is always negative for the ‘proportionate’ mixing scenario, meaning that the overall mixing is disassortative (Supplementary Fig. S2). For the disassortative mixing scenario, the combination of groups which are given priority affects the value of *Q* (Supplementary Fig. S2). Combination (b), where PB and DD are given priority, shows the most even reductions in *Q* (when compared with ‘proportionate’ mixing) across the range explored here, and is used for disassortative mixing in all subsequent reported results and figures (i.e. priority is given to minimising within-group mixing among PB and DD in setting mixing patterns). For each individual parameter combination, Q always progressively decreases for the mixing patterns in the following order: maximum assortative, setting plausible, proportionate, disassortative (data not shown).

#### 3.1.2 Effect of mixing scenario upon $R_{0}$

**Fig. 1:**
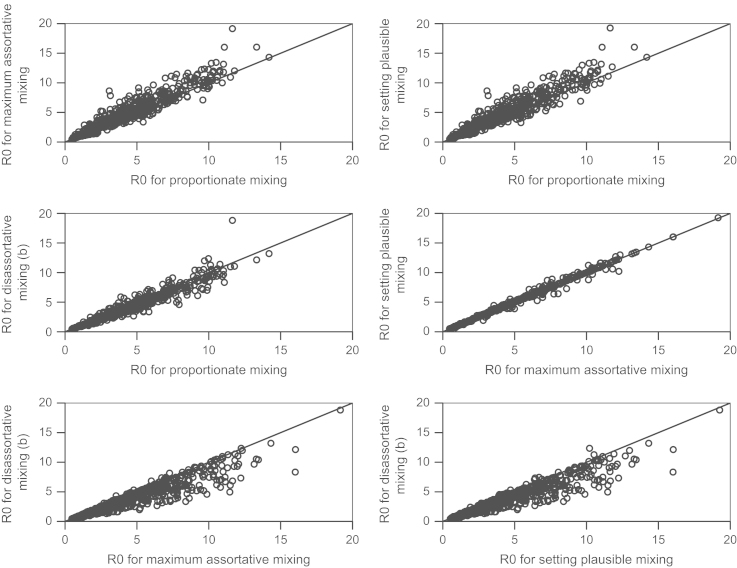
$R_{0}$**for the different mixing scenarios.**$R_{0}$ for each scenario is plotted against each other scenario in turn. Note that only one form of disassortative mixing is shown—giving priority to the PB and DD groups. The diagonal line shows equivalence.

$R_{0}$ tends to be similar for the proportionate and disassortative mixing scenarios (Fig. 1). $R_{0}$ is very similar for the maximum assortative and setting plausible mixing scenarios (Fig. 1). $R_{0}$ tends to be greater for maximum assortative and setting plausible mixing than for either proportionate or disassortative mixing, although a few exceptions are seen. A smaller range in $R_{0}$ was seen when parameters were drawn from triangular or normal distributions due to less frequent sampling of extreme parameter values (maximum $R_{0}$ ~10 for normal distributions, ~12 for triangular and ~19 for uniform distributions), but the patterns observed remained the same.

#### 3.1.3 Effect of mixing scenario upon equilibrium HIV prevalence

We noted that the solution profiles for the infected population for different initial conditions converge to a unique endemic equilibrium when $R_{0}$>1 and the disease-free equilibrium when $R_{0}$<1 (showing the existence of a forward bifurcation). Although we did not analytically derive conditions for the existence of the endemic equilibrium, the solution profiles for the infected population using different initial conditions show that the model system has a unique endemic equilibrium when $R_{0}$>1.

Equilibrium HIV prevalence is similar between mixing scenarios at higher HIV prevalences (>25%) (data not shown). At lower prevalences, maximum assortative or setting plausible mixing tend to raise the equilibrium prevalence. With maximum assortative or setting plausible mixing, epidemics sometimes occur that would not in the proportionate or disassortative scenarios. More rarely, an HIV epidemic does not occur for maximum assortative or setting plausible mixing when it does for the proportionate or disassortative scenarios (data not shown).

#### 3.1.4 Relationships between $R_{0}$, *Q* and equilibrium HIV prevalence

Across all of the different mixing scenarios, $R_{0}$ showed a weak positive association with *Q* (Supplementary Fig. S3 column 1), which was only statistically significant for proportionate and setting plausible mixing. Weak but statistically significant positive associations were seen between equilibrium prevalence and *Q* (Supplementary Fig. S3 column 2), with the strongest associations seen for the proportionate mixing scenario. A distinctive and strong relationship is seen between the equilibrium prevalence and $R_{0}$, with equilibrium prevalence 0 when $R_{0}$ is less than 1 and rising to a plateau as $R_{0}$ increases above 1 (Supplementary Fig. S3 column 3). Interestingly, this broadly follows the relationship between $R_{0}$ and endemic prevalence (*p*=1−1/$R_{0}$) that is found for simple epidemic models.

#### 3.1.5 Contribution of each group to incidence at equilibrium

The proportion of new infections generated by each group when HIV is at endemic equilibrium varies substantially across the different parameter sets. Over all the parameter sets and mixing scenarios, PB tend to cause the greatest proportion of infections (median 41%, 95% credible interval 13–79%), followed by DD (median 33%, 95% CrI 7–71%), with KH responsible for the fewest (median 20%, 95% CrI 5–54%). This differs somewhat from the relative sizes of each group: at equilibrium HIV prevalence, PB make up on average 68% of the MSM population, DD 20% and KH 12%. This pattern remains true for each mixing scenario, and there is no clear relationship between mixing scenario and the proportion of infections coming from each group. For all mixing scenarios, as $R_{0}$ increases, the proportion of new cases coming from PB increases on average, and the proportion coming from DD decreases; the proportion coming from KH decreases except when setting plausible mixing is used, when it tends to increase with higher $R_{0}$.

### 3.2 Analyses using more complex model, fitted to data

#### 3.2.1 Implications of the mixing scenario for patterns of mixing and HIV prevalence for fits to an HIV epidemic model

When separate model fits were found using each mixing scenario, more fits were found with increasing levels of disassortative mixing (maximum assortative scenario: 105 fits; setting plausible: 172; proportionate: 305; disassortative: 550). Some differences were seen between the posterior parameter distributions of the fits for the different mixing scenarios, particularly in the DD population size and the number of sex acts for each identity group (Supplementary Fig. S4).

**Fig. 2:**
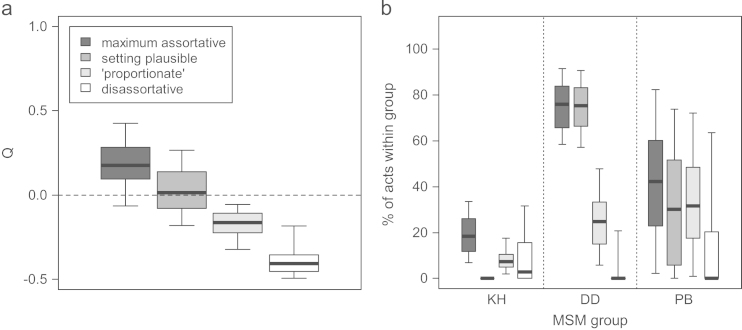
**(a) Overall mixing (**Q**) and (b) degree of mixing within groups, for fits found using different mixing scenarios**. Q and degree of mixing within groups are shown at their 2006 values. The heavy line in the middle of each box indicates the median, the limits of the box the 25th and 75th percentiles, and the whiskers the 2.5th and 97.5th percentiles. Mixing scenarios presented in decreasing order of assortativeness. Number of fits: assortative 105, setting plausible 171, proportionate 305, disassortative 550.

Comparing the fits obtained using the different mixing scenarios, *Q* and the group-specific level of within-group mixing both varied substantially between scenarios (Fig. 2). Despite these differences, the fitted HIV prevalence for each group was very similar for the different mixing scenarios, both at the fitted time points (shown for 2006, Fig. 3a) and for future predictions (shown for 2020, Fig. 3b).

#### 3.2.2 Implications of mixing scenario for predicted impact of a targeted PrEP intervention

**Fig. 4:**
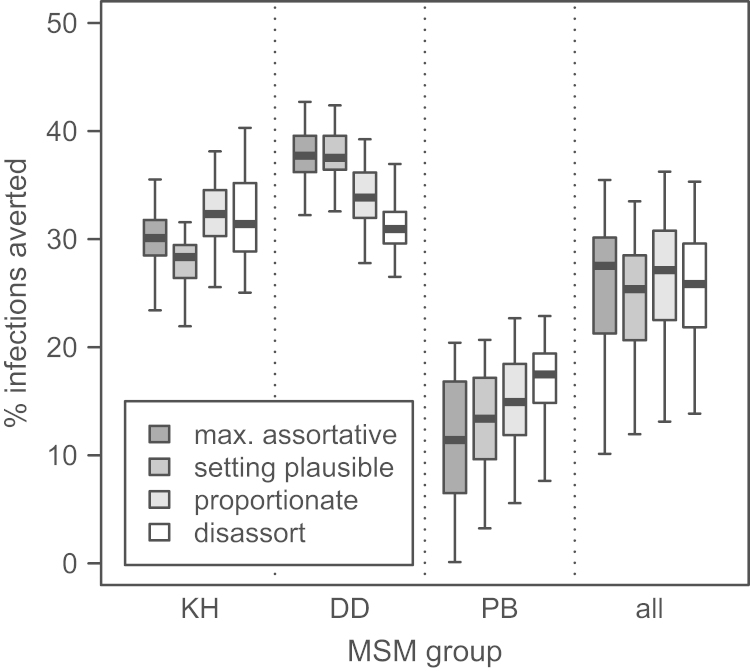
**Percentage of infections averted during a 15-year PrEP intervention with 42% effectiveness and 60% coverage of the KH and DD groups (no treatment of PB group), for different mixing scenarios**. The heavy line in the middle of each box indicates the median, the limits of the box the 25th and 75th percentiles, and the whiskers the 2.5th and 97.5th percentiles. Mixing scenarios presented in decreasing order of assortativeness. Number of fits: assortative 105, setting plausible 171, proportionate 305, disassortative 550.

**Fig. 5:**
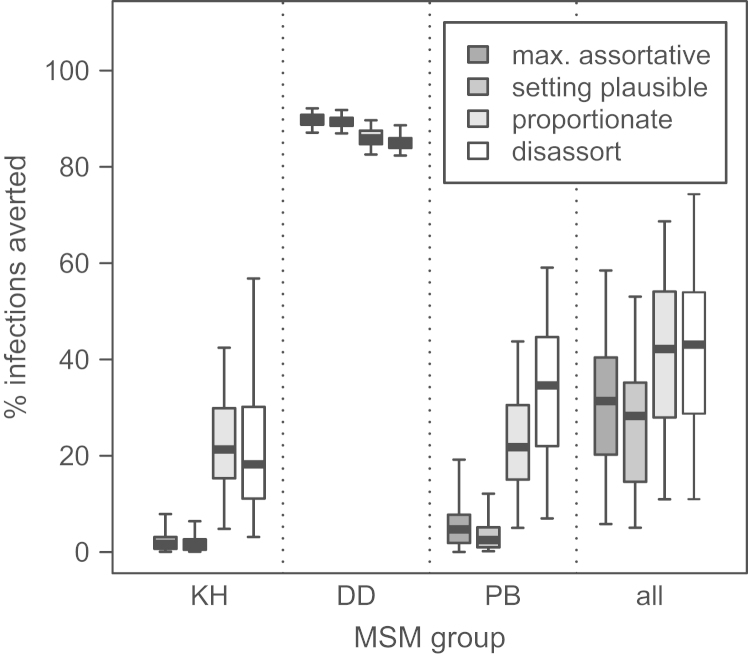
**Percentage of infections averted during a 15-year PrEP intervention with 92% effectiveness and 90% coverage of DD group alone (no treatment of KH or PB groups), for different mixing scenarios**. The heavy line in the middle of each box indicates the median, the limits of the box the 25th and 75th percentiles, and the whiskers the 2.5th and 97.5th percentiles. Mixing scenarios presented in decreasing order of assortativeness. Number of fits: assortative 105, setting plausible 171, proportionate 305, disassortative 550.

When a plausible PrEP intervention was modelled – 42% effectiveness, 60% coverage, targeted at KH and DD – using different mixing patterns gave somewhat different estimates for the group-specific impact for all three groups, particularly the DD and PB, but the overall impact in the whole MSM population was very similar (Fig. 4; Table 1). The estimated percentage of infections averted after 15 years of PrEP for this intervention strategy ranged from a median of 25.4% (IQR 20.6–28.5%) for setting plausible mixing to 27.5% (IQR 21.3–30.1%) for assortative mixing. For this same targeting strategy, no great difference in the predicted overall impact was seen for lower (30%) PrEP coverage or for an optimistic PrEP strategy (92% effectiveness, 90% coverage; Table 1). Targeting only KH gave similar results—the overall impact was similar for the different mixing scenarios, for all of the PrEP strategies (Table 1). When only DD were targeted, substantial differences were seen in the overall impact for each of the intervention strategies, most dramatically for the optimistic intervention strategy (92% effectiveness, 90% coverage; Fig. 5, Table 1)—the overall impact ranged from 28.3% of infections averted for setting plausible mixing (IQR 14.6–35.2%) to 43.1% for disassortative mixing (IQR 28.7–54.0%). If only PB were targeted, differences were also seen in the overall impact under the optimistic intervention strategy (Table 1)—the overall impact ranged from 50.7% of infections averted for assortative mixing to 63.9% for disassortative. When PrEP was given at the same coverage to all three groups, very little difference was seen in either the group-specific or the total population impact.

#### 3.2.3 Effective reproductive number for PrEP interventions

The effective reproductive number, R, varied considerably across the different parameter sets. Just before the introduction of PrEP (with condom use at 20% below its peak level), R was around 1 (median R=1.02, IQR 0.92–1.14 for proportionate mixing), and this was very similar for the different mixing scenarios. Increasingly effective PrEP interventions reduced R further: when PrEP was given to all MSM groups, R was a median 0.89 for a 42% effective PrEP intervention with 30% coverage, reduced to 0.76 with 60% coverage, and to 0.17 for a 92% effective PrEP intervention with 90% coverage, and was not affected by the mixing scenario. R was higher when only a single group was targeted, and varied somewhat between different mixing patterns for the high coverage PrEP intervention.

#### 3.2.4 Analyses using model with constant population size

Analyses using the assumption of a constant population size gave similar results for the variability in Q and prevalence values (although it predicted higher levels of prevalence – around 2.3-fold higher – in 2020 than the model with variable population size). The effective reproduction number and impact of a targeted PrEP intervention were all of a similar magnitude for both versions of the model.

## 4 Discussion

This analysis has shown that a range of different mixing patterns are consistent with observed behavioural data from MSM in Bangalore, and that these different mixing patterns can have a large effect on epidemic potential for scenarios that otherwise have the same risk behaviour. However, despite these effects, all possible mixing patterns are consistent with available HIV prevalence trends from Bangalore, and have little effect on projected future HIV prevalence trends. In addition, the different mixing patterns have little effect upon the predicted impact of an untargeted PrEP intervention, but can affect the impact of a targeted PrEP intervention, especially if targeted at groups with versatile behaviour (rather than those mainly taking a fixed role in anal sex).

It is difficult from this analysis to draw conclusions about the ‘true’ mixing pattern for this population, since very different scenarios, particularly with regard to the extent of mixing within the versatile group, are compatible with group-specific HIV prevalence data. The proportionate mixing matrix considered here tends to give rise to a partially disassortative mixing pattern (median of *Q* −0.16, IQR −0.22 to −0.11), with the insertive group having the most within-group mixing, and very little within-group mixing amongst versatile MSM; the extent of within-group mixing in this matrix is heavily impacted by the relative sizes of the different groups as well as their preference for insertive and receptive sex, and the insertive group is considerably larger than the other groups. In contrast, the ‘setting plausible’ matrix by design gives considerable within-group mixing for versatile MSM, and tends to result in more assortative population-level mixing (median of *Q* 0.00, IQR −0.07 to 0.11).

In line with epidemiological theory, the mixing scenarios which gave the most assortative mixing tended to give a higher $R_{0}$ and were more likely to give rise to an HIV epidemic (Garnett et al., 1992; Vynnycky and White, 2010). However, using the different mixing scenarios in a model which was then fit to HIV prevalence data showed that although they give rise to considerably different mixing patterns, each can be used to fit the model to available HIV prevalence data, and all give very similar projections for future HIV prevalence trends (although posterior parameter distributions differ substantially). Additionally, if these fitted models are used to project the impact of an intervention given to all groups, then the mixing scenario has little effect on the intervention impact. If the intervention is targeted at particular identity groups, however, the mixing scenario may affect the predicted impact. KH and DD are the most likely groups to be targeted in this setting, since they are more easily identified (and more likely to self-identify) as MSM. PB are less likely to be targeted, since they tend to be harder to reach, and are a larger, lower-risk and more ‘hidden’ population than either the KH or the DD (Prudden et al., 2012), so that although the model predicts differences in the overall impact between the different mixing scenarios when PB alone are targeted, this is unlikely to be a targeting strategy that is considered in this setting. For the most likely targeting strategies in this setting, where KH or KH and DD together are targeted, the overall impact of the intervention is unaffected by the mixing scenario. However, when DD alone are targeted, differences in the overall impact are predicted, and so if this strategy is considered, our modelling suggests that MSM mixing patterns could have a large effect on the intervention impact.

PrEP interventions targeting DD had the greatest variation in predicted impact by mixing scenario, due to DD having the greatest variation in within-group mixing across the different mixing scenarios. These differences occur because DD are versatile in their sexual role behaviour, having large quantities of both receptive and insertive sex, and so they can either have a large proportion of acts with other DD under the more assortative mixing scenarios, or many acts with members of other MSM groups for the more disassortative mixing scenarios.

The *Q* metric which we have used to measure the extent of overall mixing has some limitations: it only takes into account within-group mixing, weights the different groups equally (Keeling and Rohani, 2008; Newman, 2003), and its lower bound varies with the number of groups considered (Doherty et al., 2009). However, as our mixing matrices here all contained the same number of groups, the *Q* metric can be used to make direct comparisons between matrices.

This analysis highlights the importance of collecting MSM sexual mixing data in this setting, especially for the versatile DD, to improve our understanding of the likely impact of targeted interventions and to better guide resource allocation, and for identifying the most suitable mixing matrix for future modelling of this population. Until such data become available, this analysis suggests that any of the mixing scenarios used here would be suitable for use in MSM models which are used for estimating prevalence trends or the impact of non-targeted interventions, but caution should be exercised if the models are being used to predict the impact of targeted interventions in these or similar MSM populations.

## Figures and Tables

**Fig. 3:**
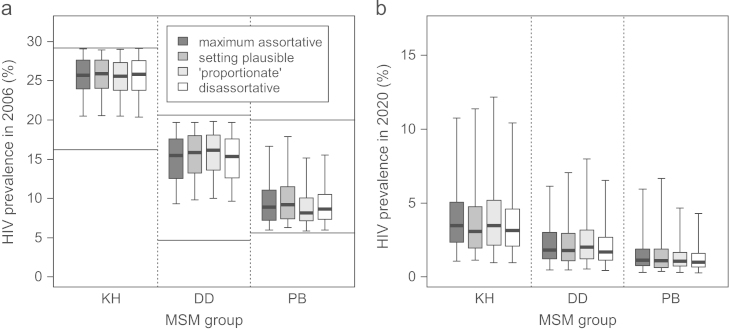
**HIV prevalence in (a) 2006 and (b) 2020, for different groups for fits found using different mixing scenarios.** The heavy line in the middle of each box indicates the median, the limits of the box the 25th and 75th percentiles, and the whiskers the 2.5th and 97.5th percentiles. Mixing scenarios presented in decreasing order of assortativeness. Number of fits: assortative 105, setting plausible 171, proportionate 305, disassortative 550. The fitting bounds for HIV prevalence in 2006 are shown (horizontal lines). Note the different *y*-axis scales for (a) and (b).

**Table 1:** **Median percentage of infections averted in whole MSM population for different PrEP intervention strategies.** Eff.=effectiveness (efficacy×adherence). Text in bold highlights where the absolute difference in impact between the mixing scenarios is 10% or more or where the relative difference in impact ((max–min)/min) is 20% or greater.

**Intervention**			**Mixing scenario**				**Greatest difference in impact between scenarios**	
**Target group**	**Eff.**	**Coverage**	**Maximum assortative**	**Setting plausible**	**Proportionate**	**Disassortative**	**Absolute**	**Relative**
KH	42	30	6.7	7.2	6.4	6.3	0.9	14.9
KH	42	60	13.3	14.3	12.8	12.5	1.8	14.6
KH	92	90	38.9	44.0	40.4	39.5	5.1	13.0
DD	42	30	6.6	6.1	7.4	7.0	1.3	**24.7**
DD	42	60	12.5	11.4	14.5	13.9	3.0	**26.4**
DD	92	90	31.4	28.3	42.2	43.1	**14.8**	**52.4**
KH+DD	42	30	14.3	13.0	14.1	13.2	1.2	9.5
KH+DD	42	60	27.5	25.4	27.1	25.8	2.1	8.5
KH+DD	92	90	73.3	70.6	73.6	73.5	2.9	4.2
PB	42	30	9.6	11.4	10.1	11.5	1.8	19.1
PB	42	60	18.4	21.9	19.5	22.4	4.0	**21.9**
PB	92	90	50.7	58.6	54.4	63.9	**13.2**	**26.1**
KH+DD+PB	42	30	22.9	22.9	23.0	23.0	0.1	0.6
KH+DD+PB	42	60	41.9	41.8	42.0	41.8	0.2	0.5
KH+DD+PB	92	90	92.2	92.3	92.3	92.2	0.1	0.1
