# Supplementary material for: Who mixes with whom among men who have sex with men? Implications for modelling the HIV epidemic in southern India
Source: J Theor Biol. 2014 Aug 21;355(100):140–50. doi: 10.1016/j.jtbi.2014.04.005 (PMC4064301; doi:10.1016/j.jtbi.2014.04.005)
Supplement: Supplementary file 1 — Supplementary data [file mmc1.docx]

# *Supplementary Information for*

**Who mixes with whom among men who have sex with men? Implications for modelling the HIV epidemic in southern India.**

Mitchell KM, Foss AM, Prudden HJ, Mukandavire Z, Pickles M, Williams JR, Johnson HC, Ramesh BM, Washington R, Isac S, Rajaram S, Phillips AE, Bradley J, Alary M, Moses S, Lowndes CM, Watts CH, Boily M-C, Vickerman P

# Supplementary Text 1: Mixing matrices

## Mixing matrix with maximum assortative mixing

The maximum number of receptive acts that members of group $i$ can have with other members of the same group, group $i$, is derived first, as the smaller of their total number of insertive and receptive acts, $\min\left( {Ins}_{i},{Rec}_{i} \right)$, where $\min$ = minimum. Any remaining receptive acts for group $i$ are assumed to be with one of the other groups. The total number of receptive acts that members of group $i$ have with members of group $j$ is the minimum of the number of receptive acts group $i$ has remaining and the number of insertive acts that group $j$has remaining after the maximum number of insertive acts have been assigned with their own group, $\min\left( \left( {Rec}_{i}-\min\left( {Ins}_{i},{Rec}_{i} \right) \right),\left( {Ins}_{j}-\min\left( {Ins}_{j},{Rec}_{j} \right) \right) \right)$. The number of insertive acts group $i$ has with group $j$ is assumed to be the same as the number of receptive acts that group $j$ has with group $i$. The order in which the different identity groups are considered does not affect the calculated mixing. The probability that, if an individual in group $i$ has a receptive act, it is with an individual in group $j$ $\left( \rho_{Rec,i,j} \right)$, is calculated as the number of receptive acts that group $i$ has with group $j$, divided by the total number of receptive acts for group $i$ (${Rec}_{i}$), with a similar calculation used for the insertive act probabilities $\left( \rho_{Ins,x,y} \right)$.

The full mixing matrix for receptive acts $\boldsymbol{\rho}_{Rec}$ with elements $\rho_{Rec,ij}$ for ($i,j$)= KH, PB and DD is as follows:

$$\boldsymbol{\rho}_{Rec}\boldsymbol{=}\left( \begin{matrix} \rho_{Rec,KH,KH} & \rho_{Rec,KH,PB} & \rho_{Rec,KH,DD} \\ \rho_{Rec,PB,KH} & \rho_{Rec,PB,PB} & \rho_{Rec,PB,DD} \\ \rho_{Rec,DD,KH} & \rho_{Rec,DD,PB} & \rho_{Rec,DD,DD} \end{matrix} \right)$$

Where

$$\left\{ \begin{matrix} \begin{matrix} \rho_{Rec,KH,KH}=\left( \min\left( {Ins}_{KH},{Rec}_{KH} \right) \right)/{{Rec}_{KH},} \\ \rho_{Rec,KH,PB}=\left( \min\left( \left( {Rec}_{KH}-\min\left( {Ins}_{KH},{Rec}_{KH} \right) \right),\left( {Ins}_{PB}-\min\left( {Ins}_{PB},{Rec}_{PB} \right) \right) \right) \right)/{{Rec}_{KH},} \end{matrix} \\ \rho_{Rec,KH,DD}=\left( \min\left( \left( {Rec}_{KH}-\min\left( {Ins}_{KH},{Rec}_{KH} \right) \right),\left( {Ins}_{DD}-\min\left( {Ins}_{DD},{Rec}_{DD} \right) \right) \right) \right)/{{Rec}_{KH},} \\ \begin{matrix} \rho_{Rec,PB,KH}=\left( \min\left( \left( {Rec}_{PB}-\min\left( {Ins}_{PB},{Rec}_{PB} \right) \right),\left( {Ins}_{KH}-\min\left( {Ins}_{KH},{Rec}_{KH} \right) \right) \right) \right)/{{Rec}_{PB},} \\ \rho_{Rec,PB,PB}=\left( \min\left( {Ins}_{PB},{Rec}_{PB} \right) \right)/{{Rec}_{PB},} \\ \begin{matrix} \rho_{Rec,PB,DD}=\left( \min\left( \left( {Rec}_{PB}-\min\left( {Ins}_{PB},{Rec}_{PB} \right) \right),\left( {Ins}_{DD}-\min\left( {Ins}_{DD},{Rec}_{DD} \right) \right) \right) \right)/{{Rec}_{PB},} \\ \rho_{Rec,DD,KH}=\left( \min\left( \left( {Rec}_{DD}-\min\left( {Ins}_{DD},{Rec}_{DD} \right) \right),\left( {Ins}_{KH}-\min\left( {Ins}_{KH},{Rec}_{KH} \right) \right) \right) \right)/{{Rec}_{DD},} \\ \begin{matrix} \rho_{Rec,DD,PB}=\left( \min\left( \left( {Rec}_{DD}-\min\left( {Ins}_{DD},{Rec}_{DD} \right) \right),\left( {Ins}_{PB}-\min\left( {Ins}_{PB},{Rec}_{PB} \right) \right) \right) \right)/{{Rec}_{DD}, \mathrm{and}} \\ \rho_{Rec,DD,DD}=\left( \min\left( {Ins}_{DD},{Rec}_{DD} \right) \right)/{{Rec}_{DD}}. \end{matrix} \end{matrix} \end{matrix} \end{matrix} \right.$$

In a similar fashion, the mixing matrix for insertive acts is given by:

$$\boldsymbol{\rho}_{Ins}\boldsymbol{=}\left( \begin{matrix} \rho_{Ins,KH,KH} & \rho_{Ins,KH,PB} & \rho_{Ins,KH,DD} \\ \rho_{Ins,PB,KH} & \rho_{Ins,PB,PB} & \rho_{Ins,PB,DD} \\ \rho_{Ins,DD,KH} & \rho_{Ins,DD,PB} & \rho_{Ins,DD,DD} \end{matrix} \right)$$

Where

$$\left\{ \begin{matrix} \begin{matrix} \rho_{Ins,KH,KH}=\left( \min\left( {Ins}_{KH},{Rec}_{KH} \right) \right)/{{Ins}_{KH},} \\ \rho_{Ins,KH,PB}=\left( \min\left( \left( {Rec}_{PB}-\min\left( {Ins}_{PB},{Rec}_{PB} \right) \right),\left( {Ins}_{KH}-\min\left( {Ins}_{KH},{Rec}_{KH} \right) \right) \right) \right)/{{Ins}_{KH},} \end{matrix} \\ \rho_{Ins,KH,DD}=\left( \min\left( \left( {Rec}_{DD}-\min\left( {Ins}_{DD},{Rec}_{DD} \right) \right),\left( {Ins}_{KH}-\min\left( {Ins}_{KH},{Rec}_{KH} \right) \right) \right) \right)/{{Ins}_{KH}}, \\ \begin{matrix} \rho_{Ins,PB,KH}=\left( \min\left( \left( {Rec}_{KH}-\min\left( {Ins}_{KH},{Rec}_{KH} \right) \right),\left( {Ins}_{PB}-\min\left( {Ins}_{PB},{Rec}_{PB} \right) \right) \right) \right)/{{Ins}_{PB},} \\ \rho_{Ins,PB,PB}=\left( \min\left( {Ins}_{PB},{Rec}_{PB} \right) \right)/{{Ins}_{PB}}, \\ \begin{matrix} \rho_{Ins,PB,DD}=\left( \min\left( \left( {Rec}_{DD}-\min\left( {Ins}_{DD},{Rec}_{DD} \right) \right),\left( {Ins}_{PB}-\min\left( {Ins}_{PB},{Rec}_{PB} \right) \right) \right) \right)/{{Ins}_{PB},} \\ \rho_{Ins,DD,KH}=\left( \min\left( \left( {Rec}_{KH}-\min\left( {Ins}_{KH},{Rec}_{KH} \right) \right),\left( {Ins}_{DD}-\min\left( {Ins}_{DD},{Rec}_{DD} \right) \right) \right) \right)/{{Ins}_{DD},} \\ \begin{matrix} \rho_{Ins,DD,PB}=\left( \min\left( \left( {Rec}_{PB}-\min\left( {Ins}_{PB},{Rec}_{PB} \right) \right),\left( {Ins}_{DD}-\min\left( {Ins}_{DD},{Rec}_{DD} \right) \right) \right) \right)/{{Ins}_{DD},}\mathrm{and} \\ \rho_{InsDD,DD}=\left( \min\left( {Ins}_{DD},{Rec}_{DD} \right) \right)/{{Ins}_{DD}}. \end{matrix} \end{matrix} \end{matrix} \end{matrix} \right.$$

## ‘Setting plausible’ mixing matrix (with assortative DD mixing and disassortative KH/PB mixing)

The number of receptive acts that DD have with other DD is maximised, as the smaller of the total number of insertive and receptive acts for DD $\left( \min\left( {Ins}_{DD},{Rec}_{DD} \right) \right)$. The number of receptive acts that KH have with PB is maximised, by calculating the smaller of the total number of receptive acts for KH and insertive acts for PB: $\left( \min\left( {Ins}_{PB},{Rec}_{KH} \right) \right)$. If the KH have any remaining receptive acts, then as many as possible of the remaining KH receptive acts are assumed to be with DD instead of with other KH (whichever is smaller of the number of remaining receptive acts for KH, and the number of remaining insertive acts for DD) : $\min\left( \left( {Ins}_{DD}-\left( \min\left( {Ins}_{DD},{Rec}_{DD} \right) \right) \right),\left( {Rec}_{KH}-\left( \min\left( {Ins}_{PB},{Rec}_{KH} \right) \right) \right) \right)$. Any KH receptive acts still remaining are assumed to be with other KH. The number of receptive acts that PB have with KH is maximised in the same way, with any remaining PB receptive acts being distributed to DD and then to other PB. DD receptive acts with KH are calculated as the smaller of the remaining receptive acts for DD and the remaining insertive acts for KH: $\min\left( \left( {Rec}_{DD}-\left( \min\left( {Ins}_{DD},{Rec}_{DD} \right) \right) \right),\left( {Ins}_{KH}-\left( \min\left( {Ins}_{KH},{Rec}_{PB} \right) \right) \right) \right)$. Similarly, DD receptive acts with PB are calculated as: $\min\left( \left( {Rec}_{DD}-\left( \min\left( {Ins}_{DD},{Rec}_{DD} \right) \right) \right),\left( {Ins}_{PB}-\left( \min\left( {Ins}_{PB},{Rec}_{KH} \right) \right) \right) \right)$. Numbers of insertive acts and the mixing probabilities $\rho_{rec,x,y}$ and $\rho_{ins,x,y}$ are calculated in the same way as in the assortative matrix.

The full mixing matrices are:

$$\boldsymbol{\rho}_{Rec}\boldsymbol{=}\left( \begin{matrix} \rho_{Rec,KH,KH} & \rho_{Rec,KH,PB} & \rho_{Rec,KH,DD} \\ \rho_{Rec,PB,KH} & \rho_{Rec,PB,PB} & \rho_{Rec,PB,DD} \\ \rho_{Rec,DD,KH} & \rho_{Rec,DD,PB} & \rho_{Rec,DD,DD} \end{matrix} \right)$$

Where

$$\left\{ \begin{matrix} \rho_{Rec,KH,KH}=\left( {Rec}_{KH}-\min\left( {Ins}_{PB},{Rec}_{KH} \right)-\min\left( {Ins}_{DD}-\min\left( {Ins}_{DD},{Rec}_{DD} \right),{Rec}_{KH}-\min\left( {Ins}_{PB},{Rec}_{KH} \right) \right) \right)/{{Rec}_{KH}}, \\ \rho_{Rec,KH,PB}=\left( \min\left( {Ins}_{PB},{Rec}_{KH} \right) \right)/{{Rec}_{KH},} \\ \begin{matrix} \rho_{Rec,KH,DD}=\left( \min\left( {Ins}_{DD}-\min\left( {Ins}_{DD},{Rec}_{DD} \right),{Rec}_{KH}-\min\left( {Ins}_{PB},{Rec}_{KH} \right) \right) \right)/{{Rec}_{KH}}, \\ \rho_{Rec,PB,KH}=\left( \min\left( {Ins}_{KH},{Rec}_{PB} \right) \right)/{{Rec}_{PB},} \\ \begin{matrix} \rho_{Rec,PB,PB}=\left( {Rec}_{PB}-\min\left( {Ins}_{KH},{Rec}_{PB} \right)-\min\left( {Ins}_{DD}-\min\left( {Ins}_{DD},{Rec}_{DD} \right),{Rec}_{PB}-\min\left( {Ins}_{KH},{Rec}_{PB} \right) \right) \right)/{{Rec}_{PB}}, \\ \rho_{Rec,PB,DD}=\left( \min\left( {Ins}_{DD}-\min\left( {Ins}_{DD},{Rec}_{DD} \right),{Rec}_{PB}-\min\left( {Ins}_{KH},{Rec}_{PB} \right) \right) \right)/{{Rec}_{PB},} \\ \begin{matrix} \rho_{Rec,DD,KH}=\left( \min\left( \left( {Rec}_{DD}-\min\left( {Ins}_{DD},{Rec}_{DD} \right) \right),\left( {Ins}_{KH}-\min\left( {Ins}_{KH},{Rec}_{PB} \right) \right) \right) \right)/{{Rec}_{DD},} \\ \rho_{Rec,DD,PB}=\left( \min\left( \left( {Rec}_{DD}-\min\left( {Ins}_{DD},{Rec}_{DD} \right) \right),\left( {Ins}_{PB}-\min\left( {Ins}_{PB},{Rec}_{KH} \right) \right) \right) \right)/{{Rec}_{DD},}\mathrm{and} \\ \rho_{Rec,DD,DD}=\left( \min\left( {Ins}_{DD},{Rec}_{DD} \right) \right)/{{Rec}_{DD}}. \end{matrix} \end{matrix} \end{matrix} \end{matrix} \right.$$

$$\boldsymbol{\rho}_{Ins}\boldsymbol{=}\left( \begin{matrix} \rho_{Ins,KH,KH} & \rho_{Ins,KH,PB} & \rho_{Ins,KH,DD} \\ \rho_{Ins,PB,KH} & \rho_{Ins,PB,PB} & \rho_{Ins,PB,DD} \\ \rho_{Ins,DD,KH} & \rho_{Ins,DD,PB} & \rho_{Ins,DD,DD} \end{matrix} \right)$$

Where

$$\left\{ \begin{aligned} \begin{matrix} \begin{matrix} \begin{matrix} \rho_{Ins,KH,KH}=\left( {Rec}_{KH}-\min\left( {Ins}_{PB},{Rec}_{KH} \right)-\min\left( {Ins}_{DD}-\min\left( {Ins}_{DD},{Rec}_{DD} \right),{Rec}_{KH}-\min\left( {Ins}_{PB},{Rec}_{KH} \right) \right) \right)/{{Ins}_{KH}}, \\ \rho_{Ins,KH,PB}=\left( \min\left( {Ins}_{KH},{Rec}_{PB} \right) \right)/{{Ins}_{KH},} \\ \rho_{Ins,KH,DD}=\left( \min\left( {Rec}_{DD}-\min\left( {Ins}_{DD},{Rec}_{DD} \right),{Ins}_{KH}-\min\left( {Ins}_{KH},{Rec}_{PB} \right) \right) \right)/{{Ins}_{KH}}, \end{matrix} \\ \rho_{Ins,PB,KH}=\left( \min\left( {Ins}_{PB},{Rec}_{KH} \right) \right)/{{Ins}_{PB},} \\ \rho_{Ins,PB,PB}=\left( {Rec}_{PB}-\min\left( {Ins}_{KH},{Rec}_{PB} \right)-\min\left( {Ins}_{DD}-\min\left( {Ins}_{DD},{Rec}_{DD} \right),{Rec}_{PB}-\min\left( {Ins}_{KH},{Rec}_{PB} \right) \right) \right)/{{Ins}_{PB},} \end{matrix} \\ \rho_{Ins,PB,DD}=\left( \min\left( {Rec}_{DD}-\min\left( {Ins}_{DD},{Rec}_{DD} \right),{Ins}_{PB}-\min\left( {Ins}_{PB},{Rec}_{KH} \right) \right) \right)/{{Ins}_{PB},} \\ \rho_{Ins,DD,KH}=\left( \min\left( \left( {Ins}_{DD}-\min\left( {Ins}_{DD},{Rec}_{DD} \right) \right),\left( {Rec}_{KH}-\min\left( {Ins}_{PB},{Rec}_{KH} \right) \right) \right) \right)/{{Ins}_{DD},} \end{matrix} \\ \rho_{Ins,DD,PB}=\left( \min\left( \left( {Ins}_{DD}-\min\left( {Ins}_{DD},{Rec}_{DD} \right) \right),\left( {Rec}_{PB}-\min\left( {Ins}_{KH},{Rec}_{PB} \right) \right) \right) \right)/{{Ins}_{DD},} \\ \rho_{Ins,DD,DD}=\left( \min\left( {Ins}_{DD},{Rec}_{DD} \right) \right)/{{Ins}_{DD}} \end{aligned} \right.$$

## Mixing matrix with ‘proportionate’ mixing

Proportionate mixing is frequently used to determine mixing in models stratified by sexual activity (Gupta et al., 1989). Here, it is used to calculate the probability that an individual in group $i$ has a receptive act with an individual in group $j$, **given** that the person in group $i$ has a receptive act; this probability is calculated as the number of insertive acts offered by group $j$ divided by the number of insertive acts offered by the whole population.

The probability that, if an individual in group $i$ has a receptive act, it is with an individual in group $j$, is given by

$$\rho_{Rec,i,j}=\frac{{Ins}_{j}}{{Ins}_{i}+{Ins}_{j}+{Ins}_{k}}$$

where ${Ins}_{k}$ is the number of insertive acts per year for group $k$.

The insertive mixing probability is calculated in a similar way:

$$\rho_{Ins,i,j}=\frac{{Rec}_{j}}{{Rec}_{i}+{Rec}_{j}+{Rec}_{k}}$$

where ${Rec}_{k}$ is the number of receptive acts per year for group $k$.

The full mixing matrices are:

$${\boldsymbol{\rho}_{Rec}}\boldsymbol{=}\left( \begin{matrix} \frac{{Ins}_{KH}}{{Ins}_{KH}+{Ins}_{PB}+{Ins}_{DD}} & \frac{{Ins}_{PB}}{{Ins}_{KH}+{Ins}_{PB}+{Ins}_{DD}} & \frac{{Ins}_{DD}}{{Ins}_{KH}+{Ins}_{PB}+{Ins}_{DD}} \\ \frac{{Ins}_{KH}}{{Ins}_{KH}+{Ins}_{PB}+{Ins}_{DD}} & \frac{{Ins}_{PB}}{{Ins}_{KH}+{Ins}_{PB}+{Ins}_{DD}} & \frac{{Ins}_{DD}}{{Ins}_{KH}+{Ins}_{PB}+{Ins}_{DD}} \\ \frac{{Ins}_{KH}}{{Ins}_{KH}+{Ins}_{PB}+{Ins}_{DD}} & \frac{{Ins}_{PB}}{{Ins}_{KH}+{Ins}_{PB}+{Ins}_{DD}} & \frac{{Ins}_{DD}}{{Ins}_{KH}+{Ins}_{PB}+{Ins}_{DD}} \end{matrix} \right)$$

$${\boldsymbol{\rho}_{Ins}}\boldsymbol{=}\left( \begin{matrix} \frac{{Rec}_{KH}}{{Rec}_{KH}+{Rec}_{PB}+{Rec}_{DD}} & \frac{{Rec}_{PB}}{{Rec}_{KH}+{Rec}_{PB}+{Rec}_{DD}} & \frac{{Rec}_{DD}}{{Rec}_{KH}+{Rec}_{PB}+{Rec}_{DD}} \\ \frac{{Rec}_{KH}}{{Rec}_{KH}+{Rec}_{PB}+{Rec}_{DD}} & \frac{{Rec}_{PB}}{{Rec}_{KH}+{Rec}_{PB}+{Rec}_{DD}} & \frac{{Rec}_{DD}}{{Rec}_{KH}+{Rec}_{PB}+{Rec}_{DD}} \\ \frac{{Rec}_{KH}}{{Rec}_{KH}+{Rec}_{PB}+{Rec}_{DD}} & \frac{{Rec}_{PB}}{{Rec}_{KH}+{Rec}_{PB}+{Rec}_{DD}} & \frac{{Rec}_{DD}}{{Rec}_{KH}+{Rec}_{PB}+{Rec}_{DD}} \end{matrix} \right)$$

## Mixing matrix with disassortative mixing

The algorithm gives priority to maximising the disassortative mixing for two groups, with the third group taking any remaining acts. The algorithm was applied three times for each parameter set, prioritising a different pair of groups each time.

For the groups which are prioritised ($i$ and $j$), the number of receptive acts that group $i$ have with group $j$ is maximised, calculated as the smaller of the total number of receptive acts for group $i$ and the number of insertive acts for group $j$*,* $\min\left( {Ins}_{j},{Rec}_{i} \right)$. As many as possible of any remaining receptive acts for group $i$ are given to group $k$: $\min\left( {Ins}_{k}, {Rec}_{i}-\min\left( {Ins}_{j},{Rec}_{i} \right) \right)$. Any remaining receptive acts for group $i$ are assumed to be with other members of group $i$: ${Rec}_{i}-min\left( {Ins}_{j},{Rec}_{i} \right)-min\left( {Ins}_{k}, {Rec}_{i}-\min\left( {Ins}_{j},{Rec}_{i} \right) \right)$. Receptive acts for group $j$are distributed in the same way. For group $k$, the total number of receptive acts they have with any other group is calculated as the number of remaining insertive acts available from each other group. Numbers of insertive acts and the mixing probabilities $\rho_{Rec,i,j}$ and $\rho_{Ins,i,j}$ are calculated in the same way as in the assortative matrix.

The full mixing matrices are given with the KH and PB groups prioritised.

$$\boldsymbol{\rho}_{Rec}\boldsymbol{=}\left( \begin{matrix} \rho_{Rec,KH,KH} & \rho_{Rec,KH,PB} & \rho_{Rec,KH,DD} \\ \rho_{Rec,PB,KH} & \rho_{Rec,PB,PB} & \rho_{Rec,PB,DD} \\ \rho_{Rec,DD,KH} & \rho_{Rec,DD,PB} & \rho_{Rec,DD,DD} \end{matrix} \right)$$

Where

$$\left\{ \begin{aligned} \begin{matrix} \begin{matrix} \begin{matrix} \rho_{Rec,KH,KH}=\left( {Rec}_{KH}-\min\left( {Ins}_{PB},{Rec}_{KH} \right)-\min\left( {Ins}_{DD},{Rec}_{KH}-\min\left( {Ins}_{PB},{Rec}_{KH} \right) \right) \right)/{{Rec}_{KH},} \\ \rho_{Rec,KH,PB}=\left( \min\left( {Ins}_{PB},{Rec}_{KH} \right) \right)/{{Rec}_{KH},} \\ \rho_{Rec,KH,DD}=\left( \min\left( {Ins}_{DD},{Rec}_{KH}-\min\left( {Ins}_{PB},{Rec}_{KH} \right) \right) \right)/{{Rec}_{KH},} \end{matrix} \\ \rho_{Rec,PB,KH}=\left( \min\left( {Ins}_{KH},{Rec}_{PB} \right) \right)/{{Rec}_{PB},} \\ \rho_{Rec,PB,PB}=\left( {Rec}_{PB}-\min\left( {Ins}_{KH},{Rec}_{PB} \right)-\min\left( {Ins}_{DD},{Rec}_{PB}-\min\left( {Ins}_{KH},{Rec}_{PB} \right) \right) \right)/{{Rec}_{PB},} \end{matrix} \\ \rho_{Rec,PB,DD}=\left( \min\left( {Ins}_{DD},{Rec}_{PB}-\min\left( {Ins}_{KH},{Rec}_{PB} \right) \right) \right)/{{Rec}_{PB},} \\ \rho_{Rec,DD,KH}=\left( {Ins}_{KH}-\min\left( {Ins}_{KH},{Rec}_{PB} \right)-\left( {Rec}_{KH}-\min\left( {Ins}_{PB},{Rec}_{KH} \right)-\min\left( {Ins}_{DD},{Rec}_{KH}-\min\left( {Ins}_{PB},{Rec}_{KH} \right) \right) \right) \right)/{{Rec}_{DD},} \end{matrix} \\ \rho_{Rec,DD,PB}=\left( {Ins}_{PB}-\min\left( {Ins}_{PB},{Rec}_{KH} \right)-\left( {Rec}_{PB}-\min\left( {Ins}_{KH},{Rec}_{PB} \right)-\min\left( {Ins}_{DD},{Rec}_{PB}-\min\left( {Ins}_{KH},{Rec}_{PB} \right) \right) \right) \right)/{{Rec}_{DD}, \mathrm{and}} \\ \rho_{Rec,DD,DD}=\left( {Ins}_{DD}-\min\left( {Ins}_{DD},{Rec}_{KH}-\min\left( {Ins}_{PB},{Rec}_{KH} \right) \right)-\min\left( {Ins}_{DD},{Rec}_{PB}-\min\left( {Ins}_{KH},{Rec}_{PB} \right) \right) \right)/{{Rec}_{DD}}. \end{aligned} \right.$$

$$\boldsymbol{\rho}_{Ins}\boldsymbol{=}\left( \begin{matrix} \rho_{Ins,KH,KH} & \rho_{Ins,KH,PB} & \rho_{Ins,KH,DD} \\ \rho_{Ins,PB,KH} & \rho_{Ins,PB,PB} & \rho_{Ins,PB,DD} \\ \rho_{Ins,DD,KH} & \rho_{Ins,DD,PB} & \rho_{Ins,DD,DD} \end{matrix} \right)$$

Where

$$\left\{ \begin{aligned} \begin{matrix} \begin{matrix} \begin{matrix} \rho_{Ins,KH,KH}=\left( {Rec}_{KH}-\min\left( {Ins}_{PB},{Rec}_{KH} \right)-\min\left( {Ins}_{DD},{Rec}_{KH}-\min\left( {Ins}_{PB},{Rec}_{KH} \right) \right) \right)/{{Ins}_{KH}}, \\ \rho_{Ins,KH,PB}=\left( \min\left( {Ins}_{KH},{Rec}_{PB} \right) \right)/{{Ins}_{KH},} \\ \rho_{Ins,KH,DD}=\left( {Ins}_{KH}-\min\left( {Ins}_{KH},{Rec}_{PB} \right)-\left( {Rec}_{KH}-\min\left( {Ins}_{PB},{Rec}_{KH} \right)-\min\left( {Ins}_{DD},{Rec}_{KH}-\min\left( {Ins}_{PB},{Rec}_{KH} \right) \right) \right) \right)/{{Ins}_{KH},} \end{matrix} \\ \rho_{Ins,PB,KH}=\left( \min\left( {Ins}_{PB},{Rec}_{KH} \right) \right)/{{Ins}_{PB},} \\ \rho_{Ins,PB,PB}=\left( {Rec}_{PB}-\min\left( {Ins}_{KH},{Rec}_{PB} \right)-\min\left( {Ins}_{DD},{Rec}_{PB}-\min\left( {Ins}_{KH},{Rec}_{PB} \right) \right) \right)/{{Ins}_{PB}}, \end{matrix} \\ \rho_{Ins,PB,DD}=\left( {Ins}_{PB}-\min\left( {Ins}_{PB},{Rec}_{KH} \right)-\left( {Rec}_{PB}-\min\left( {Ins}_{KH},{Rec}_{PB} \right)-\min\left( {Ins}_{DD},{Rec}_{PB}-\min\left( {Ins}_{KH},{Rec}_{PB} \right) \right) \right) \right)/{{Ins}_{PB},} \\ \rho_{Ins,DD,KH}=\left( \min\left( {Ins}_{DD},\left( {Rec}_{KH}-\min\left( {Ins}_{PB},{Rec}_{KH} \right) \right) \right) \right)/{{Ins}_{DD},} \end{matrix} \\ \rho_{Ins,DD,PB}=\left( \min\left( {Ins}_{DD},\left( {Rec}_{PB}-\min\left( {Ins}_{KH},{Rec}_{PB} \right) \right) \right) \right)/{{Ins}_{DD},}\mathrm{and} \\ \rho_{Ins,DD,DD}=\left( {Ins}_{DD}-\min\left( {Ins}_{DD},{Rec}_{KH}-\min\left( {Ins}_{PB},{Rec}_{KH} \right) \right)-\min\left( {Ins}_{DD},{Rec}_{PB}-\min\left( {Ins}_{KH},{Rec}_{PB} \right) \right) \right)/{{Ins}_{DD}}. \end{aligned} \right.$$

## Number of sex acts

The total number of receptive acts that group $i$ has with group $j$ is calculated for each mixing scenario from the probability that an individual in group $i$ who has a receptive act has it with an individual in group $j$ ($\rho_{Rec,i,j}$) multiplied by the total number of receptive acts that group $i$ has (${Rec}_{i})$. Similarly, the number of insertive acts that group $i$ has with group $j$ is given by $\rho_{Ins,i,j}{Ins}_{i}$. When sex acts are balanced according to equations (3) and (4), then for all mixing scenarios the number of receptive acts that group $i$ has with group $j$ is the same as the number of insertive acts that group $j$has with group $i$, meeting the constraints necessary for this type of mixing function (Busenberg and Castillo-Chavez, 1991).

## Overall mixing probabilities

The overall level of mixing between different groups was calculated by summing the number of insertive and receptive acts that group $i$ have with group $j$, and dividing by the total number of sex acts that group $i$ has: $\left( \rho_{Rec,i,j}{Rec}_{i}+\rho_{Ins,i,j}{Ins}_{i} \right)/{N_{i}c_{i}}$.This was used to produce a matrix of overall mixing probabilities.

# Supplementary Tables

**Supplementary Table S1. Parameters used in the modelling analysis.** The parameter value and range used are given, together with the source for each estimate.

| **Parameter symbol** | **Description** | **Mode^1^ (mean where different)** | **Range^2^** | **Standard deviation^3^** | **Units** | **Source** |
| --- | --- | --- | --- | --- | --- | --- |
| **ALL MODELS** | |  |  |  |  |  |
| $\boldsymbol{x}_{\boldsymbol{KH}}$ | Proportion of KH sex acts which are insertive | 0.1 | 0.03-0.17 | 0.02 | - | SBS survey 2006 |
| $\boldsymbol{x}_{\boldsymbol{DD}}$ | Proportion of DD sex acts which are insertive | 0.37 | 0.28-0.46 | 0.026 | - | SBS survey 2006 |
| $\boldsymbol{x}_{\boldsymbol{PB}}$ | Proportion of PB sex acts which are insertive | 0.75 | 0.50-1.00 | 0.071 | - | SBS survey 2006, wider plausible range used to account for possible bias in the sample |
| $\boldsymbol{\mu}_{\boldsymbol{KH}}$ | Rate at which KH leave the population | 0.05645 | 0.0282-0.0847 | 0.0081 | Person^-1^ year^-1^ | IBBA survey 2006 |
| $\boldsymbol{\mu}_{\boldsymbol{DD}}$ | Rate at which DD leave the population | 0.05745 | 0.0287-0.0862 | 0.0082 | Person^-1^ year^-1^ | IBBA survey 2006 |
| $\boldsymbol{\mu}_{\boldsymbol{PB}}$ | Rate at which PB leave the population | 0.05085 | 0.0254-0.0763 | 0.0073 | Person^-1^ year^-1^ | IBBA survey 2006 |
| $\boldsymbol{N}_{\boldsymbol{KH}}$ | Total number of KH | 9339 | 6,226-12,452 | 889.4 | People | NGO estimate |
| $\boldsymbol{N}_{\boldsymbol{DD}}$ | Total number of DD | 16603 | 2,075-31,130 | 4150.6 | People | Estimate relative to $N_{KH}$ |
| $\boldsymbol{N}_{\boldsymbol{PB}}$ | Total number of PB | [calculated in model] | 2,000-120,000  [permitted range] |  | People | Calculated from sizes of total MSM population and of KH and DD groups |
| $\boldsymbol{N}_{\boldsymbol{TOT}}$ | Total MSM population size | [calculated in model] | 8,501-122,600  [permitted range] |  | People | PBS in nearby districts |
| $\boldsymbol{c}_{\boldsymbol{KH}}$ | Average number of sex acts per person per year for KH | 278 | 114-442 | 46.9 | Person^-1^ year^-1^ | IBBA survey 2006 |
| $\boldsymbol{c}_{\boldsymbol{DD}}$ | Average number of sex acts per person per year for DD | 208 | 104-312 | 29.7 | Person^-1^ year^-1^ | IBBA survey 2006 |
| $\boldsymbol{c}_{\boldsymbol{PB}}$ | Average number of sex acts per person per year for PB | 105 | 2-208 | 29.4 | Person^-1^ year^-1^ | IBBA survey 2006, lower bound reduced to allow for possible bias in the sample |
| **INITIAL MODEL** | |  |  |  |  |  |
| $1/\alpha$ | Duration from HIV infection to developing AIDS | 7.8 (9.6) | 4.2-15 | 1.54 | Years | Lui et al. (1988) |
| $\boldsymbol{\beta}_{\boldsymbol{rec}}$ | Per-act risk of acquiring HIV through receptive anal sex | 0.00775 | 0.0015-0.014 | 0.0018 | Per act | Baggaley et al.  (2010) |
| $\kappa$ | Relative risk of acquiring HIV from insertive vs. receptive anal sex | 0.14 | 0.03-0.25 | 0.031 | - | Jin et al. (2010), Pinkerton et al. (2000), Vittinghoff et al. (1999) |
| **FULL MODEL** | |  |  |  |  |  |
| $\alpha$ | Rate at which those in pre-AIDS stage develop AIDS | 0.63 | Fixed value |  | Person^-1^ year^-1^ | Hollingsworth et al. (2008) |
| ${1/\gamma}_{1}$ | Duration of acute HIV stage | 0.175 | 0.125-0.225 |  | Years | Hollingsworth et al. (2008) |
| $\gamma_{2}$ | Rate of progression from chronic HIV to pre-AIDS stage | 0.165 | Fixed value |  | Person^-1^ year^-1^ | Hollingsworth et al. (2008), Lui et al. (1988) |
| $\beta_{rec}^{2}$ | Per-act risk of acquiring HIV through receptive anal sex with a partner with chronic HIV | 0.00775 | 0.0015-0.014 |  | Per act | Baggaley et al. (2010) |
| $\kappa$ | Relative risk of acquiring HIV from insertive vs. receptive anal sex | 0.14 | 0.03-0.25 |  | - | Jin et al. (2010), Pinkerton et al. (2000), Vittinghoff et al. (1999) |
| $\nu_{1}$ | Relative infectiousness of individual with acute HIV compared with chronic HIV | 11.65 | 4.5-18.8 |  | - | Boily et al. (2009) |
| $\nu_{3}$ | Relative infectiousness of individual in pre-AIDS stage compared with chronic HIV | 8.2 | 4.5-11.9 |  | - | Boily et al. (2009) |
| $\pi_{0}$ | Proportion of acts in which condoms are used correctly at baseline | 12.5 | 0-25 |  | % | Analysis of data from IBBA survey 2006; Lowndes et al. (2010) |
| $\pi_{1}$ | Proportion of acts in which condoms are used correctly in 2006 | 87.7 | 76.7-98.7 |  | % | IBBA surveys 2006 & 2009 |
| $\Delta_{c}$ | Absolute increase in condom use (*C*) prior to 2006 | 7.6 | 6.7-8.6 |  | Year-^1^ | Analysis of data from IBBA survey 2006; Lowndes et al. (2010) |
| $e_{C}$ | Efficacy of condoms against HIV | 0.84 | 0.74-0.94 |  | Per act | Weller and Davis-Beaty (2002), allowing for 7.8% condom breakage reported in the IBBA survey 2009 |
| $\tilde{p}$ | Coverage of PrEP – proportion of MSM using PrEP | 0.6 | 0.3, 0.6 or 0.9 |  | - | Scenarios explored |
| $f_{P}$ | Effectiveness (efficacy x adherence) of PrEP against HIV | 0.42 | 0.42 or 0.92 |  | Per act | Grant et al. (2011; 2010) |
| $T_{HIV}$ | Year in which HIV introduced into the MSM population | 1986 | 1981-1990 |  | Calendar year | Simoes et al. (1993) |
| $\sigma_{KH}$ | Initial HIV prevalence in KH | 2 | 0-4 |  | % | Same range as for female sex workers (Arora et al., 2004) |
| $\sigma_{DD}$ | Initial HIV prevalence in DD | 2 | 0-4 |  | % | Same range as for female sex workers (Arora et al., 2004) |
| $\sigma_{PB}$ | Initial HIV prevalence in PB | 2 | 0-4 |  | % | Same range as for female sex workers (Arora et al., 2004) |
| **HIV prevalence fitting ranges:** | | |  |  |  |  |
| $\boldsymbol{HIV}_{\boldsymbol{KH,2006}}$ | HIV prevalence among KH in 2006 | - | 16.2-29.2 |  | % | IBBA survey 2006 |
| $\boldsymbol{HIV}_{\boldsymbol{DD,2006}}$ | HIV prevalence among DD in 2006 | - | 5.6-20.0 |  | % | IBBA survey 2006 |
| $\boldsymbol{HIV}_{\boldsymbol{PB,2006}}$ | HIV prevalence among PB in 2006 | - | 4.7-20.6 |  | % | IBBA survey 2006 |
| $\boldsymbol{HIV}_{\boldsymbol{KH,2009}}$ | HIV prevalence among KH in 2009 | - | 16.2-28.8 |  | % | IBBA survey 2009 |
| $\boldsymbol{HIV}_{\boldsymbol{DD,2009}}$ | HIV prevalence among DD in 2009 | - | 6.8-17.4 |  | % | IBBA survey 2009 |
| $\boldsymbol{HIV}_{\boldsymbol{PB,2009}}$ | HIV prevalence among PB in 2009 | - | 4.4-21.8 |  | % | IBBA survey 2009 |

^1^Value specified as mode for triangular distribution and mean for normal distribution (numbers given in brackets were used for the mean in the normal distribution)

^2^Range specified for the uniform and triangular distributions

^3^Standard deviation specified for the normal distribution

# Supplementary figures

**Supplementary Figure S1. Numerical analysis of endemic equilibrium.** (a) HIV prevalence in DD with different DD initial conditions for $R_{0}$ = 1.18, setting plausible mixing; (b) HIV prevalence in DD with different DD initial conditions for $R_{0}$ = 0.77, setting plausible mixing. The same qualitative patterns (a single endemic equilibrium for $R_{0}$>1, disease-free equilibrium for $R_{0}$<1) were seen for all groups for all of the mixing matrices, for all parameter combinations and initial conditions investigated.

(a)

(b)

**Supplementary Figure S2.** $Q$ **(measure of overall assortativity of mixing) for the different disassortative mixing scenarios.** For each disassortative scenario, $Q$ is plotted against the proportionate mixing scenario. The three different versions of disassortative mixing give priority to minimising within-group mixing for (a) KH and PB, (b) PB and DD, and (c) KH and DD. The diagonal line shows equivalence.

**Supplementary Figure S3. Relationship between** $R_{0}$**,** $Q$ **and equilibrium HIV prevalence for each mixing scenario.** The best-fit linear regression line and R^2^ values are added for the relationships between $R_{0}$ and $Q$, and equilibrium prevalence and $Q$. On the plots of prevalence against $R_{0}$, the relationship between $R_{0}$ and 100*(1-1/$R_{0}$) is shown by the black line, and the R^2^ value for the relationship between prevalence and 1/$R_{0}$ is shown and marked with an asterisk. The different mixing scenarios shown are: (a) maximum assortative; (b) setting plausible; (c) proportionate; (d) disassortative, where PB and DD are given priority in determining mixing.

**Supplementary Figure S4. Posterior parameter distributions for selected parameters.** Posterior parameter distributions are shown relative to their prior distribution for each of the four mixing scenarios, for the four parameters for which the posteriors varied by more than 20% between mixing scenarios.

**References**

Arora, P., Cyriac, A., Jha, P., 2004. India's HIV-1 epidemic. CMAJ 171, 1337-1338, doi:10.1503/cmaj.045032.

Baggaley, R. F., White, R. G., Boily, M. C., 2010. HIV transmission risk through anal intercourse: systematic review, meta-analysis and implications for HIV prevention. Int J Epidemiol 39, 1048-1063, doi:10.1093/ije/dyq057.

Boily, M. C., Baggaley, R. F., Wang, L., Masse, B., White, R. G., Hayes, R. J., Alary, M., 2009. Heterosexual risk of HIV-1 infection per sexual act: systematic review and meta-analysis of observational studies. Lancet Infect Dis 9, 118-129.

Busenberg, S., Castillo-Chavez, C., 1991. A general solution of the problem of mixing of subpopulations and its application to risk- and age structured epidemic models for the spread of AIDS. IMA J Math Appl Med Biol 8, 1-29.

Grant, R., McMahan, V., Liu, A., Guanira, J., Casapia, M., Lama, J., Fernandez, T., Veloso, V., Buchbinder, S., Chariyalertsak, S., Schechter, M., Bekker, L.-G., Mayer, K., Kallas, E., Anderson, P., Amico, K. R., Glidden, D., 2011. Completed observation of the randomized placebo-controlled phase of iPrEx: daily oral FTC/TDF pre-exposure HIV prophylaxis among men and trans women who have sex with men. International AIDS Society conference, Rome.

Grant, R. M., Lama, J. R., Anderson, P. L., McMahan, V., Liu, A. Y., Vargas, L., Goicochea, P., Casapía, M., Guanira-Carranza, J. V., Ramirez-Cardich, M. E., Montoya-Herrera, O., Fernández, T., Veloso, V. G., Buchbinder, S. P., Chariyalertsak, S., Schechter, M., Bekker, L.-G., Mayer, K. H., Kallás, E. G., Amico, K. R., Mulligan, K., Bushman, L. R., Hance, R. J., Ganoza, C., Defechereux, P., Postle, B., Wang, F., McConnell, J. J., Zheng, J.-H., Lee, J., Rooney, J. F., Jaffe, H. S., Martinez, A. I., Burns, D. N., Glidden, D. V., 2010. Preexposure chemoprophylaxis for HIV prevention in men who have sex with men. N Engl J Med 363, 2587-2599, doi:10.1056/NEJMoa1011205.

Gupta, S., Anderson, R. M., May, R. M., 1989. Networks of sexual contacts - implications for the pattern of spread of HIV. AIDS 3, 807-817.

Hollingsworth, T. D., Anderson, R. M., Fraser, C., 2008. HIV-1 transmission, by stage of infection. J Infect Dis 198, 687-693, doi:10.1086/590501.

Jin, F. Y., Jansson, J., Law, M., Prestage, G. P., Zablotska, I., Imrie, J. C. G., Kippax, S. C., Kaldor, J. M., Grulich, A. E., Wilson, D. P., 2010. Per-contact probability of HIV transmission in homosexual men in Sydney in the era of HAART. AIDS 24, 907-913, doi:10.1097/QAD.0b013e3283372d90.

Lowndes, C. M., Alary, M., Verma, S., Demers, E., Bradley, J., Jayachandran, A. A., Ramesh, B. M., Moses, S., Adhikary, R., Mainkar, M. K., 2010. Assessment of intervention outcome in the absence of baseline data: 'reconstruction' of condom use time trends using retrospective analysis of survey data. Sex Transm Infect 86, I49-I55, doi:10.1136/sti.2009.038802.

Lui, K. J., Darrow, W. W., Rutherford, G. W., 1988. A model-based estimate of the mean incubation period for AIDS in homosexual men. Science 240, 1333-1335, doi:10.1126/science.3163848.

Pinkerton, S. D., Abramson, P. R., Kalichman, S. C., Catz, S. L., Johnson-Masotti, A. P., 2000. Secondary HIV transmission rates in a mixed-gender sample. Int J STD AIDS 11, 38-44, doi:10.1258/0956462001914887.

Simoes, E. A. F., Babu, P. G., Jeyakumari, H. M., John, T. J., 1993. The initial detection of human immunodeficiency virus-1 and its subsequent spread in prostitutes in Tamil Nadu, India. J Acquir Immune Defic Syndr Hum Retrovirol 6, 1030-1034.

Vittinghoff, E., Douglas, J., Judson, F., McKirnan, D., MacQueen, K., Buchbinder, S. P., 1999. Per-contact risk of human immunodeficiency virus transmission between male sexual partners. Am J Epidemiol 150, 306-311.

Weller, S. C., Davis-Beaty, K., 2002. Condom effectiveness in reducing heterosexual HIV transmission. Cochrane Database Syst Rev, CD003255.
